# Context-Aware Semantic Localization with Adaptive Sensor Fusion Under Adverse Conditions

**DOI:** 10.3390/s26041328

**Published:** 2026-02-19

**Authors:** Jun-Hyeon Choi, Dong-Su Seo, Ye-Chan An, Tae-Wook Eum, Jin-Ho Kim, Gi-Hyeon Kwon, Tae-Yong Kuc, Jeong-Won Pyo

**Affiliations:** 1 Department of Electrical and Computer Engineering, College of Information and Communication Engineering, Sungkyunkwan University, Suwon 16419, Republic of Korea; choijunhyeon@g.skku.edu (J.-H.C.);; 2 Department of Intelligent Robotics, College of Engineering, Sungkyunkwan University, Suwon 16419, Republic of Korea; 3 DXR Co., Ltd., R&D Center, Seoul 01411, Republic of Korea

**Keywords:** semantic localization, semantic map, context-aware, ontology, sensor fusion

## Abstract

To achieve Level 4 and above autonomous driving, vehicle localization must remain accurate and reliable under diverse real-world conditions, including complex traffic scenarios, environmental changes, and partial sensor failures. Conventional localization approaches primarily rely on geometric consistency among multi-sensor observations, which can produce physically or contextually implausible pose estimates when sensor reliability degrades or observations become ambiguous. This paper proposes a semantic localization framework that integrates ontology-based semantic reasoning directly into the localization process. The proposed approach reformulates localization as a context-aware constraint selection problem guided by semantic consistency among objects, places, and vehicle poses. By evaluating logical and contextual validity at the hypothesis generation stage, semantically invalid pose hypotheses are eliminated early, and only situation-appropriate semantic constraints are selectively applied during optimization. As a result, compared to the localization system without semantic rules, the proposed framework achieves an average reduction of approximately 35.6% in mean localization error and 47.0% in maximum localization error across both longitudinal and lateral directions. Specifically, the framework supports structured multi-sensor fusion by selectively using sensor information semantically relevant to the driving context. Through this semantics-driven hypothesis reduction, the system reduces computational complexity while enhancing localization robustness and accuracy, particularly under sensor degradation and dynamic environmental conditions. The design of the semantic reasoning structure is also adaptable to cooperative perception scenarios, such as V2V-based information sharing.

## 1. Introduction

As autonomous driving advances to Level 4, where vehicles operate without human intervention, the demands on perception, decision-making, and control systems are fundamentally increasing. In particular, localization is a core component, as accurate vehicle positioning is essential for reliable path planning, decision-making, and collision avoidance.

However, real-world conditions differ significantly from ideal. Complex traffic scenarios, adverse weather, sensor degradation, and frequent GNSS outages in urban canyons or tunnels reduce sensor reliability and increase localization uncertainty. In addition, long-term operation inevitably involves sensor faults and hardware failures. Therefore, a robust localization framework is needed that can withstand adverse conditions.

Traditional localization systems estimate vehicle poses using individual sensors such as GNSS, IMU, cameras, and LiDAR, or their geometric fusion, by optimizing numerical consistency between sensor observations and map data. Although these methods achieve high accuracy under stable conditions, their reliance on purely geometric and numerical criteria leads to fundamental limitations when sensor reliability degrades or environmental conditions change abruptly. In particular, the lack of logical and semantic context allows physically or contextually implausible estimates, such as vehicles located outside lanes or in non-navigable regions, to be selected if geometric errors are minimized, resulting in error accumulation and context-inconsistent localization during long-term operation.

To overcome these limitations, localization must incorporate information beyond numerical consistency. Semantic information encodes the meaningful structure of road environments, including relationships among roads, lanes, intersections, and objects, enabling the early rejection of physically or contextually invalid pose hypotheses. By evaluating contextual validity at the constraint-generation stage rather than adjusting sensor reliability only after errors occur, semantic-based localization suppresses logically inconsistent information, even under sensor faults or transient observation anomalies.

In our previous studies, semantic knowledge and contextual information were applied to task planning [1,2] and task allocation [3,4], enabling robots to select situation-appropriate actions beyond purely geometric representations. By incorporating object-place relationships and task context, semantic-based planning improved the robustness and reliability of robotic task execution in complex environments. However, these efforts were primarily confined to high-level planning, while localization still relied on sensor measurements and geometric consistency.

This paper extends semantic modeling and reasoning from task planning to localization. Ontology-based semantic reasoning eliminates logically or contextually invalid pose hypotheses, while a context-aware action reasoning mechanism selectively applies relevant semantic constraints based on the driving context. In addition, although autonomous vehicles receive abundant information from multiple heterogeneous sensors, the proposed framework leverages semantic knowledge to selectively use only context-relevant sensor information, enabling effective, structured multi-sensor fusion. Through this semantic-driven hypothesis reduction and selective sensor utilization, the proposed approach reduces the optimization search space and computational burden while improving localization accuracy and robustness under incomplete sensor information and dynamic real-world conditions. Furthermore, the semantic reasoning structure naturally supports extensibility to cooperative perception scenarios, such as V2V-based information sharing, by enabling semantically consistent integration of external observations.

- **Semantic redefinition of localization:** We redefine vehicle localization as a context-aware constraint selection problem by integrating ontology-based semantic reasoning beyond geometric optimization.
- **Semantic hypothesis reduction for robust localization:** Logically invalid pose hypotheses are pre-emptively eliminated through semantic consistency evaluation among objects, places, and poses.
- **Situation-adaptive constraint selection via reasoning:** A context-aware action reasoning mechanism selectively applies semantic constraints to the localization graph.
- **Semantic-guided multi-sensor fusion and cooperative extensibility:** The proposed framework enables effective multi-sensor fusion by adaptively utilizing context-relevant sensor information through semantic reasoning and provides a scalable foundation for cooperative perception, such as V2V-based.

## 2. Related Work

### 2.1. Traditional Localization and SLAM

Traditional localization and SLAM estimate poses by optimizing geometric consistency between sensor observations and map representations using features such as keypoints, edges, or scan alignments [5,6]. Vision-based [7,8], LiDAR-based [9,10], and tightly coupled visual-inertial [11] or LiDAR-inertial [12,13] methods have demonstrated high accuracy under stable sensing conditions and well-structured environments.

Recently, learning-based localization and SLAM approaches have gained increasing attention, driven by advances in deep neural networks and improved computational capabilities [14,15]. These methods leverage convolutional or transformer-based architectures to regress poses, perform place recognition, or learn robust feature representations directly from data. In parallel, hardware advancements have enabled highly optimized geometric pipelines, such as GPU-accelerated scan matching and dense registration methods, to operate at unprecedented speeds, further improving real-time performance [16,17].

In addition, as discussed in Section 2.1, learning-based methods leveraging deep neural networks have been explored to enhance multi-sensor fusion, enabling robust feature extraction, cross-modal association, and adaptive weighting of sensor measurements in complex real-world environments [18,19].

Despite their demonstrated accuracy, purely geometric localization and SLAM methods exhibit fundamental limitations in complex real-world environments. Repetitive man-made structures, texture-less road surfaces, dynamic agents, and adverse weather conditions significantly reduce geometric discriminability, often resulting in ambiguous data associations and unstable optimization. More critically, these methods rely solely on numerical consistency and lack semantic or contextual reasoning, making them unable to judge the physical or logical validity of estimated poses. As a consequence, geometrically optimal yet physically implausible solutions can be selected, exposing an inherent vulnerability of purely geometry-driven pipelines when deployed in unconstrained real-world scenarios.

### 2.2. Multi-Sensor Fusion-Based Localization

To overcome the limitations of single-sensor localization, multi-sensor fusion approaches combine complementary modalities such as GNSS, IMU, cameras, and LiDAR. By exploiting redundancy across heterogeneous sensors, these systems improve robustness against individual sensor failures and enhance pose estimation accuracy [20,21]. Sensor fusion frameworks commonly employ probabilistic filtering or factor graph optimization to model sensor uncertainties and achieve consistent pose estimation [22]. Such robustness and redundancy are particularly critical for safety-critical autonomous driving systems, where reliable localization under sensor degradation or failure is essential to ensure operational integrity and trustworthiness [23,24].

Recent multi-sensor fusion research has explored adaptive and learning-enhanced fusion strategies to overcome the limitations of static sensor reliability models [25]. Beyond purely learning-based approaches, several works introduce adaptive weighting mechanisms that evaluate sensor consistency, residual errors, or feature-level matching quality to dynamically adjust sensor contributions under changing environmental conditions [26,27]. These methods often leverage statistical consistency checks, residual-driven confidence estimation, or context-aware weighting functions to enhance robustness in challenging scenarios such as low illumination, perceptual degradation, or sensor degradation [28]. By relaxing the assumption of fixed noise characteristics, adaptive fusion frameworks demonstrate improved resilience in environments where traditional hand-crafted sensor models fail [29].

However, most fusion-based localization systems still lack explicit mechanisms to reason about the semantic or contextual validity of fused pose estimates [30,31]. Fusion decisions are made primarily based on numerical agreement across sensors, without verifying whether the resulting pose is compatible with high-level scene semantics or driving constraints. Consequently, even when multiple sensors exhibit strong statistical consistency, the estimated vehicle pose may violate road topology, lane boundaries, or navigability constraints. This failure to distinguish numerically consistent yet semantically invalid solutions highlights a fundamental limitation of conventional fusion paradigms, particularly in complex, safety-critical autonomous driving environments [32,33].

### 2.3. Semantic Mapping and Semantic SLAM

Semantic mapping and semantic SLAM extend traditional SLAM by augmenting geometric maps with semantic labels or object-level landmarks [34,35]. These approaches integrate object detection, segmentation, or classification results into mapping and localization pipelines, improving map interpretability, relocalization robustness, and long-term usability under environmental changes [36,37].

Recent studies have begun to move beyond using semantics as passive annotations and instead explore semantic-aware reasoning within the SLAM pipeline [38,39]. These approaches investigate the use of semantic constraints, object-level relations, and scene priors to influence data association, hypothesis pruning, and pose validation [40,41]. By incorporating semantic consistency checks or object-centric structural reasoning, such methods aim to suppress physically or contextually implausible solutions that cannot be identified through geometric optimization alone [42,43].

Additionally, recent research has increasingly explored the integration of large language models (LLMs) into robotic systems to support high-level semantic representation and reasoning [44,45]. Owing to their open-vocabulary understanding, abstract relational reasoning, and strong generalization capabilities, LLMs have emerged as a powerful alternative to traditional rule-based or ontology-driven semantic reasoning frameworks. These models are particularly effective at higher semantic layers, such as natural language instruction interpretation, task context understanding, and goal inference in unstructured environments [46,47].

Despite these advances, most semantic SLAM approaches still treat semantic information as auxiliary observations [48,49] rather than as an active mechanism for reasoning and decision-making within the localization process [50,51]. Semantic cues are typically incorporated as soft constraints or labels attached to geometric landmarks, without explicitly influencing hypothesis selection or rejection, allowing geometrically consistent but semantically invalid pose hypotheses to persist [52]. While LLMs partially mitigate this limitation through open-vocabulary reasoning and strong generalization, their role in localization remains indirect, as they usually operate on abstracted outputs from SLAM or perception modules [53]. This weak coupling with real-time perception and state estimation, together with high computational cost and the lack of explicit uncertainty modeling, limits the applicability of LLM-based approaches to real-time, closed-loop SLAM pipelines [54].

## 3. Semantic Modeling for Localization

Semantic modeling has traditionally been employed in autonomous systems to support high-level reasoning tasks such as task planning, decision-making, and action selection. In these contexts, semantic representations enable robots to interpret their environments, reason about object-place relationships, and determine feasible actions based on contextual knowledge. Such planning-oriented semantic frameworks have demonstrated improved robustness and scalability in complex, dynamic environments by enabling systems to move beyond purely geometric representations.

Building on these advantages, recent research has begun to explore integrating semantic modeling for localization, leveraging high-level contextual knowledge to improve pose estimation. By embedding semantic constraints into the localization process, systems can assess pose hypotheses not only based on geometric consistency but also on their semantic plausibility with respect to the environment. This shift allows rejection of logically invalid or contextually inconsistent poses, potentially yielding more robust and reliable localization, particularly in complex and ambiguous scenarios where purely geometric cues are insufficient.

### 3.1. Overview of Semantic Modeling Framework

Autonomous vehicles in complex road environments must reason not only about geometry but also about the semantic meaning of surrounding elements to understand situational context. Conventional representations based on low-level geometry or limited semantics are insufficient for context-aware reasoning, as they cannot explain why observations are meaningful, how elements relate to each other, or whether a candidate location is semantically feasible.

To address these limitations, high-level semantic representations that integrate structured knowledge about objects, places, and driving context are required. Such representations enable reasoning about spatial relations and contextual consistency, supporting more robust relocalization and informed decision-making in dynamic urban scenarios.

In our previous work [1,2], semantic information was mainly used for planning, allowing robots to interpret their environment and select task-appropriate actions. In contrast, this study applies semantic cues to localization, where multi-modal sensor observations and associated semantic structures are evaluated for contextual validity to weight or reject infeasible constraints during pose estimation.

This section introduces the Semantic Modeling Framework (SMF), which combines the Triplet Ontological Semantic Model (TOSM) with a semantic HD map to incorporate human-like interpretation into localization [55]. By organizing environmental information into symbolic, explicit, and implicit models, the framework extends localization beyond conventional geometric matching.

### 3.2. Semantic Knowledge Representation

Conventional HD maps provide highly accurate geometric and structural descriptions of road environments, including lane-level topology, node-link connectivity, road attributes, and registered static objects [56]. These representations are well-suited to trajectory generation and motion planning, but they are primarily designed for geometric alignment and rule-based navigation. As a result, semantic information in traditional HD maps is either implicit or weakly structured, limiting its direct applicability to localization tasks that require evaluating the contextual validity of pose hypotheses under uncertainty.

To bridge this gap, this work reinterprets HD map information from a planning-oriented semantic abstraction to a localization-aware semantic model. Instead of treating semantic elements solely as high-level descriptors for action selection, the proposed approach reorganizes map entities into object-, place-, and relation-centric representations that can actively constrain localization. By explicitly encoding which objects exist in which regions, how places are connected, and which spatial relations are semantically valid, the map provides criteria for assessing whether a candidate vehicle pose is plausible beyond geometric consistency.

#### 3.2.1. Basic Components of HD Map

An HD map is generally composed of the following key elements:

- **Node**: A node represents a meaningful point or turning point along the vehicle’s route. Each node contains geographic coordinates (latitude and longitude) and is typically defined at locations where road curvature changes, intersections occur, or where the distance between points exceeds a predefined threshold.
- **Link**: A link is a directed path that connects two nodes and represents the actual drivable route for the vehicle. Each link consists of multiple positional points, referred to as link points, which are stored as sequences of GPS or local coordinates along the path.
- **Road Type**: This attribute defines the type of road associated with a given link. Road types include general roads, tunnels, bridges, underground roads, and elevated highways.
- **Directionality**: Each link is inherently directional. The direction is explicitly specified using *FromNodeID* and *ToNodeID*, which indicate the start and end nodes of the link, respectively. The link points are stored in the order corresponding to the link’s travel direction.
- **Lane Connectivity Information**: The attributes *R_LinkID* and *L_LinkID* refer to the IDs of adjacent links on the right and left sides, respectively, when lane changes are possible from the current link.

**Figure 1:**
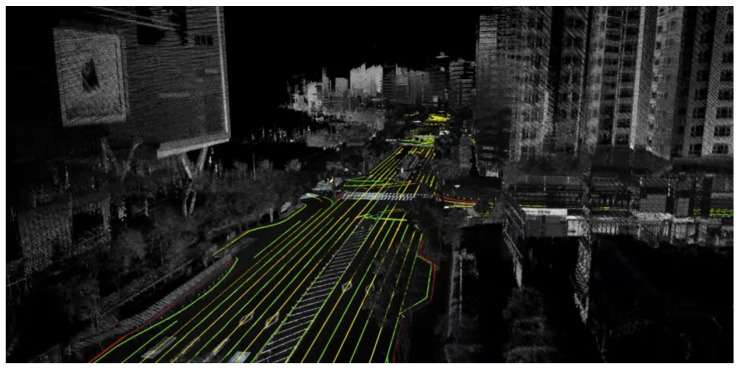
Visualization of the HD map constructed for our experimental area.

Each link contains a unique link ID, the ID of its start and end nodes, the associated road type, and a list of coordinate points known as link points. These link points represent a high-resolution sequence of positions that subdivide the drivable path along the link. Figure 1 and Table 1 and Table 2 illustrate the overall structure of the HD map and its constituent elements, respectively.

Conventional HD maps are primarily designed to support geometric alignment, route planning, and rule-based navigation by providing precise road structure information. While sufficient for trajectory generation, they lack explicitly structured semantic relations, limiting their ability to evaluate the contextual validity of pose hypotheses during localization. This limitation motivates the introduction of a Semantic HD Map that enables reasoning-based localization, as described in the following section.

#### 3.2.2. Semantic HD Map

A Semantic HD Map extends the geometric foundation of conventional HD maps by incorporating semantic information that enables reasoning-based localization. While traditional HD maps provide structural elements such as lane boundaries, road edges, node-link topologies, and road types, these geometric features alone are insufficient for Level 4 and higher autonomous driving. Vehicles must not only match geometric shapes but also interpret the meaning of surrounding objects to infer their state within the environment. To address this need, the Semantic HD Map augments the map with perceptible, semantically expressive objects, enabling a human-like understanding of spatial context.

These semantic elements incorporated into the map exhibit four key properties that directly enhance inference-based localization:

- **Object-centric:** Fixed objects such as signals, road signs, markings, reflectors, and structural elements are included as landmarks for localization.
- **Perceptibility:** Only objects detectable by on-board sensors (LiDAR, cameras) are registered.
- **Localization constraint:** Objects that exist only in specific regions act as strong constraints for narrowing the vehicle’s location.
- **Semantic reasoning:** Combining perception with semantic attributes allows inference of plausible regions, heading validity, or lane-level consistency even before graph optimization.

In this study, the Semantic HD Map is designed based on the Triplet Ontological Semantic Model (TOSM). Beyond simple coordinate representation, each object is described using symbolic, explicit, and implicit attributes, and their interrelations are encoded to support robust semantic reasoning.

**Figure 2:**
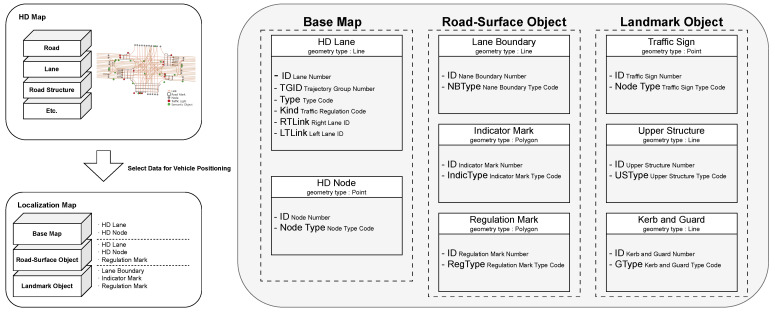
Architecture of the proposed Semantic Modeling Framework (SMF).

**Figure 3:**
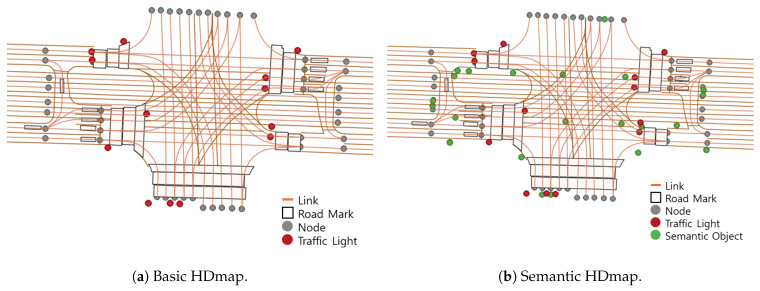
Comparison between a conventional HD map (**a**) and a semantic HD map (**b**).

Figure 2 illustrates the structure of the Semantic HD Map employed in this study. This map incorporates both geometric and semantic elements, including lane boundaries, semantic objects, and semantic categories such as bridge support types or tunnel jet fans. Figure 3 compares a conventional HD map with the semantic HD map, highlighting the enriched semantic attributes integrated into the latter.

#### 3.2.3. Semantic Object Selection Criteria

Objects registered in the Semantic HD Map are not selected arbitrarily. Only those that provide meaningful localization constraints and are observable by vehicle sensors are included.

- **Perceptibility:** Reliable detection using RGB cameras or LiDAR.
- **Localizability:** Presence restricted to specific spatial segments, reducing pose uncertainty.
- **Persistence:** Spatially fixed and temporally stable despite season, lighting, or time-of-day changes.

**Figure 4:**
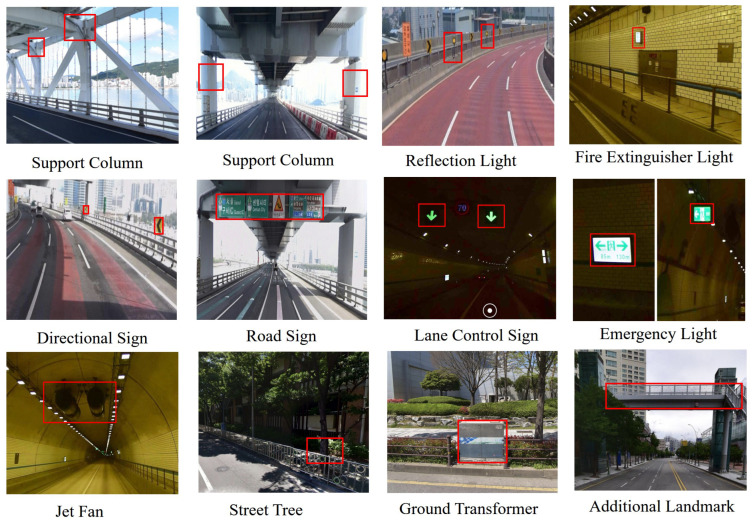
Additional semantic objects registered in the HD map for use in semantic localization.

Twelve types of objects were selected based on these principles, most of which are commonly found in tunnel interiors or beneath elevated structures where GPS signals are unreliable, as illustrated in Figure 4.

#### 3.2.4. Environmental Elements and Semantic Definitions Based on TOSM

**Figure 5:**
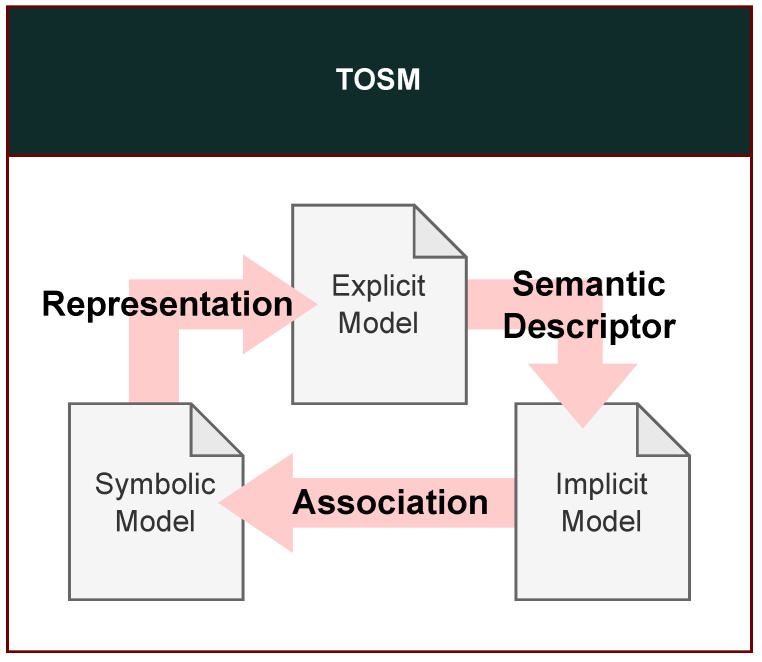
Triplet Ontological Semantic Model (TOSM).

In this section, we model the semantic elements required for vehicle localization and SLAM using the Triplet Ontological Semantic Model (TOSM) (show Figure 5). The environment is structured into three primary categories: Object, Place, and Robot, and each element is represented through explicit, implicit, and symbolic models. The following definitions specialize the original TOSM framework for use in autonomous driving and semantic HD maps, enabling environmental elements to be consumed directly by localization and SLAM.

TOSM in Figure 5 is designed as a structured representation that stores environmental elements required for semantic reasoning in a form that can be efficiently transformed into both OWL (Web Ontology Language) ontological properties [57] and PDDL planning domains. Rather than serving solely as a static semantic map, TOSM provides a unified abstraction that enables semantic information to be systematically expanded through ontology-based inference and subsequently grounded into actionable representations within a PDDL domain.

As listed in Table 3, environmental elements such as Object, Place, and Robot are organized as subclasses of a common Environmental Element superclass, allowing implicit semantic relationships to be inferred via the ontology. By extending these inferred semantics to the PDDL domain, the framework enables the system to reason about feasible and required robot actions given the current situation, thereby supporting the application of semantic factors and online information updates within the localization process. In this work, we extend the original TOSM framework [1,55], previously developed for task planning, to better support localization by explicitly coupling semantic reasoning with state estimation and map consistency.

#### 3.2.5. Object Properties for Spatial and Robot Relations

Object properties, as listed in Table 4, represent relations between environmental elements. For vehicle localization, spatial relations such as *isNextTo* and *isInsideOf* provide constraints that link objects and places. The relation *isConnectedTo* also defines constraints between places. Robot-related properties, including *isLookingAt*, *isLocatedAt*, and *isOccupiedBy*, describe how the ego-vehicle interacts with the environment in real time. Subsequently, these relations serve as constraints in the optimization process, which is detailed in Section 4.

#### 3.2.6. Datatype Properties in the Triplet Ontological Semantic Model

The Triplet Ontological Semantic Model (TOSM) represents each environmental element using structured datatype properties that encode explicit, implicit, and symbolic information. This design enables the environment to be interpreted not only as geometric entities but also as semantically meaningful elements essential for reasoning-based localization. Explicit properties refer to information directly observable through sensor processing, such as metric, geometric, and visual features. Implicit properties express inferred or relational knowledge, similar to human semantic memory, and capture how objects relate to their surroundings. Symbolic properties provide abstract identifiers that enable robots to communicate, index, and reason effectively about environmental concepts.

- **Symbolic (Conceptual Semantics):** Concept-level descriptors representing the abstract meaning or role of an entity, including identifiers, categorical labels, or regulatory attributes. These symbolic descriptors allow robots and humans to interact with the same conceptual elements in a consistent manner.
- **Explicit (Measurable Properties):** Information directly obtained from sensor modalities, such as pose, velocity, shape, boundary geometry, or image-based features (e.g., colors, keypoints). These attributes form the measurable foundation used in SLAM, map matching, and perception.
- **Implicit (Contextual Relations):** Knowledge-driven or induced relations such as spatial containment, environmental context, movability, adjacency, or lane-level complexity. These relations convey high-level semantic cues that cannot be sensed directly but are derived from domain knowledge or databases.

Each model within TOSM is represented using *classes*, *object properties*, and *datatype properties*, following OWL terminology. Environmental elements are grouped into three primary classes *Object*, *Place*, and *Robot* which are defined as subclasses of *EnvironmentalElement*. Subclasses inherit the characteristics of their superclasses, allowing hierarchical abstraction (e.g., *TunnelJetFan* inherits all attributes of *InfrastructureObject*, *Object*, and *EnvironmentalElement*). Table 5, Table 6 and Table 7 summarize these datatype properties, specialized for autonomous-driving scenarios.

These object properties and datatype properties collectively enable semantic inference for localization, allowing the system to interpret environmental elements not only by their geometric positions but also by their meanings, contexts, and interrelations. This integrated semantic structure ultimately supports more coherent and reliable estimation of the vehicle’s state within complex environments.

### 3.3. TOSM-Based Semantic Reasoning for Context-Aware Information Selection

Semantic reasoning is used to selectively extract and validate context-relevant environmental information from the TOSM representation, ensuring semantic and contextual consistency across the perception, localization, and planning modules. Environmental elements encoded in TOSM are interconnected through explicit properties, which enable rule-based reasoning to transform implicitly encoded relational knowledge into explicit semantic facts. Through this process, latent contextual relationships among places, objects, and robots are systematically inferred without requiring exhaustive enumeration in the original knowledge representation.

In addition to ontology-based inference, the proposed framework incorporates the outcomes of the PDDL-based planning process described in Section 3.4 to verify the contextual consistency of newly updated or inferred information. Semantic facts that remain consistent with the current planning context are retained, while contextually incompatible updates are discarded. This mechanism prevents semantically valid but situationally irrelevant information from propagating into downstream reasoning processes.

Based on predefined contextual conditions, rule-based semantic reasoning is further used to query only the information required for the robot’s current situation, producing a compact, on-demand semantic database. Rather than operating on the entire semantic knowledge base, this database contains only contextually admissible candidate places, objects, and relational constraints. The resulting on-demand representation is then passed to the PDDL module, which uses it directly to construct planning problem instances.

It is important to note that this semantic reasoning module is strictly responsible for contextual judgment and information filtering. It does not trigger actions, modify robot behavior, or execute control decisions; instead, it provides a semantically validated, context-consistent abstraction layer that supports localization reasoning and planning in a decoupled, modular manner.

The following SWRL rules illustrate a simple example of semantic reasoning for maintaining localization consistency.

- **Rule 1 (Localization continuity from previous place):**
  A place is considered a localization candidate only if it is connected to the robot’s previously localized place.
  (1) $$\begin{array}{cc} \; & Robot\left(?r\right)\wedge Place\left(?p_{1}\right)\wedge Place\left(?p_{2}\right)\wedge \\ \; & isLocatedAt\left(?r,?p_{1}\right)\wedge isConnectedTo\left(?p_{1},?p_{2}\right)\\ \; & \to canMoveTo(?r,?p_{2}) \end{array}$$
- **Rule 2 (Robot feasibility regulation):**
  A place is excluded if it violates the regulations.
  (2) $$\begin{array}{cc} \; & Robot\left(?r\right)\wedge Place\left(?p_{1}\right)\wedge Place\left(?p_{2}\right)\wedge \\ \; & isAlignedHeading\left(?r,?p_{2}\right)\wedge isNotRestricted\left(?p_{2}\right)\\ \; & \to isContextualTo(?r,?p_{2}) \end{array}$$
- **Rule 3 (Robot capability consistency):**
  A place is excluded if the robot cannot physically remain or maneuver within it.
  (3) $$\begin{array}{cc} \; & Robot\left(?r\right)\wedge Place\left(?p_{1}\right)\wedge Place\left(?p_{2}\right)\wedge Place\left(?p_{3}\right)\wedge \\ \; & isConnectedTo\left(?p_{1},?p_{2}\right)\wedge isConnectedTo\left(?p_{2},?p_{3}\right)\wedge \\ \; & hasDistance(?p2,?d)\wedge lessThan(?d,5.0)\\ \; & \to isReachable(?r,?p_{2}) \end{array}$$
- **Rule 4 (Semantic validity of objects):**
  A detected object is considered valid if it is semantically consistent with the current place.
  (4) $$\begin{array}{cc} \; & Robot(?r)\wedge Place(?p)\wedge Object(?o)\wedge \\ \; & isLocatedAt(?r,?p)\wedge isInsideOf(?o,?p)\wedge \\ \; & \to isCandidate(?o) \end{array}$$
- **Rule 5 (Candidate place validation by object-place consistency):**
  A place is considered a localization candidate if the detected objects are consistent with it.
  (5) $$\begin{array}{cc} \; & Robot(?r)\wedge Place(?p)\wedge Object(?o)\wedge \\ \; & isCandidate(?o)\wedge isInsideOf(?o,?p)\wedge isKeyObject(?o,?p)\\ \; & \to isLocatedAt(?r,?p) \end{array}$$
- **Rule 6 (Sensor reliability from semantic consistency):**
  A sensor is considered reliable at a place if its detected objects are semantically consistent with that place.
  (6) $$\begin{array}{ccc} \; & Robot(?r)\wedge Sensor(?s)\wedge Place(?p)\wedge \\ \; & isLocatedAt(?r,?p)\wedge isEquipped(?s,?r)\wedge \\ \; & hasConfidence(?p,?c)\wedge lessThan(?d,0.5)\; & \to isReliable(?s) \end{array}$$

Using these rule-based semantic constraints, the system performs contextual semantic judgment to validate and refine updated information, retaining only environmental elements consistent with the current situation. By filtering candidate places, objects, and sensor states based on semantic, physical, and regulatory consistency, the framework constructs a compact and context-aware representation that supports robust localization and downstream reasoning without propagating irrelevant or inconsistent information.

### 3.4. Semantic Domain Modeling for Localization

Accurate vehicle localization in complex road environments requires a representational structure that extends beyond geometric consistency to incorporate higher-level semantic cues, such as lane categories, intersection topology, object types, and region-level contextual information. These semantic cues enable the system to disambiguate visually repetitive or metrically similar scenes and to reject infeasible localization hypotheses that cannot be resolved through geometric SLAM alone.

To support this capability, the environment is modeled as a symbolic domain that explicitly captures relationships among places, objects, sensor observations, and contextual constraints. PDDL (Planning Domain Definition Language) [58] is adopted as a formal representation framework to encode these entities, their relations, and condition-dependent state transitions. Unlike conventional uses of PDDL for motion or task execution, this work leverages PDDL to infer contextual action feasibility, enabling the system to reason about which actions are currently possible, required, or disallowed given the vehicle’s semantic and spatial context.

Specifically, the PDDL-based reasoning process evaluates the current semantic state to determine admissible localization-related actions, such as hypothesis validation, constraint activation, or application of semantic factors. Based on this judgment, semantic constraints are selectively applied, and semantic information is updated in a context-consistent manner, ensuring that only feasible interpretations of the environment influence localization. In this formulation, PDDL does not trigger physical execution or motion commands; instead, it serves as a structured reasoning layer that governs how semantic evidence and constraints are incorporated into state estimation. The localization domain is therefore organized around essential components that support semantic judgment rather than explicit planning execution.

#### 3.4.1. Semantic Type Hierarchy for Localization

The localization domain requires a well-structured semantic type hierarchy that organizes all entities in the driving environment into interpretable symbolic classes. This hierarchy includes coarse HD-level regions such as road segments, intersections, and lane links, mid-level semantic places representing drivable or context-specific regions, and fine-grained unit places used for spatial occupancy reasoning. In addition, robot and sensor entities are explicitly represented to support observation-driven inference, enabling PDDL to connect raw measurements to symbolic logic. By defining this structured hierarchy, the localization system ensures that every object, place, and sensor instance can be consistently grounded onto the semantic HD map, forming the foundational layer for all subsequent predicates and reasoning actions.

Listing 1 provides a concise example of the semantic type hierarchy, illustrating how map entities such as road markings, traffic lights, semantic places, unit places, and robot-sensor instances are grounded into their corresponding symbolic classes. This mapping ensures that all physical elements in the HD map can be consistently interpreted and reasoned over during the localization process.

**Listing 1.** Example Types.(:types 
ego1 - ego 
lane1 lane2 - surface_mark 
traffic1 - traffic_light 
sign1 sign2 -  sign 
pole1 tree1 - facility 
place1 place2 - semantic_place 
unit1p1 unit1p2 - unit_place 
camera1 ladar1 - sensor 
link1 link2 intersection1 - hd_place
)

#### 3.4.2. Spatial Connectivity

Semantic localization requires the ability to reason about how different regions and objects in the environment are spatially related. To achieve this, the domain encodes core spatial relations such as adjacency, containment, and occupancy, which collectively determine whether a sensor observation is compatible with a given candidate place. These predicates allow the reasoning engine to verify that observed objects lie within the correct semantic region, ensure that no unit-level space is already occupied, and exploit lane-level properties such as permissible lane changes. Together, these spatial relations form a foundational layer that constrains and validates the localization hypotheses.

Listing 2 summarizes the core spatial predicates that encode adjacency, containment, occupancy, and lane-level constraints, forming the foundational relations required for semantic-consistency-based localization.

**Listing 2.** Example of Spatial Connectivity Predicates.
(is_connected_to ?from ?to - place)

(is_inside_of ?u - unit_place ?p - place)

(is_inside_of ?o - object ?p - place)

(is_occupied_by ?o - object ?u - unit_place)

(is_key_object ?o - object)

(can_lane_change ?l - lane)


#### 3.4.3. Vehicle State Representation

The predicates in Listing 3 define the symbolic representation of the ego vehicle’s internal and external state, covering its current location, viewing target, intended movement, occupancy relation with the environment, and high-level behavioral attributes such as goals and plans. These state descriptors enable the reasoning engine to integrate sensor observations with semantic map constraints, thereby enabling a structured update to the vehicle’s belief state throughout the localization process. By maintaining both geometric properties (pose, velocity) and symbolic properties (goal, plan), the domain supports multi-layered inference that aligns perception, intention, and semantic context [59].

**Listing 3.** Example of Vehicle State Predicates.
(is_looking_at ?e - ego ?o - object)

(is_located_at ?e - ego ?p - place)

(is_move_to ?e - ego ?p - place)

(is_occupied_by ?e - ego ?u - unit_place)

(has_plan ?e - ego)

(has_goal ?e - ego)

(pose ?e - ego)

(velocity ?e - ego)


#### 3.4.4. Sensor-Level Observation Predicates

Sensor observations are encoded as symbolic predicates that bridge the gap between continuous SLAM perception and discrete semantic reasoning [60]. By representing measurement availability, sensor placement reliability, and sensor characteristics such as frequency and coverage, the domain captures how each sensor contributes to the localization process. These predicates support filtering, hypothesis validation, and reliability-aware factor activation during reasoning.

Listing 4 defines the symbolic predicates that encode sensor measurements, reliability, and coverage characteristics, enabling observation-driven consistency checks during localization.

**Listing 4.** Example of Sensor Observation Predicates.
(is_measure_to ?s - sensor ?o - object)

(is_reliable ?s - sensor ?p - place)

(frequency ?s - sensor)

(coverage ?s - sensor)


#### 3.4.5. HD Map-Derived Semantic Constraints

Semantic constraints in the localization domain capture structural and contextual properties extracted from the HD map, including expected objects within each place, lane categories, directional attributes, local speed regulations, and sensor reliability profiles. These symbolic constraints operate as strong priors that restrict candidate locations to remain consistent with map semantics, preventing contradictory or geometrically implausible hypotheses during reasoning. By encoding these map-derived attributes, the localization process gains semantic stability, ensuring that only structurally compatible scene interpretations survive the pruning steps.

Listing 5 illustrates example predicates that encode HD map-derived semantic attributes used to constrain and interpret candidate places during localization. These constraints allow the reasoning engine to assess whether the ego vehicle’s current observations, motion context, and expected scene structure are semantically consistent with the map, thereby enabling the vehicle to understand its situational state in the environment in a symbolic and interpretable manner.

**Listing 5.** Example of HD Map Semantic Constraints.
(has_object ?p - place)

(lane_type ?p - place)

(direction ?p - place)

(sensor_reliability ?p - place)

(speed_limit ?p - place)


#### 3.4.6. Localization Reasoning Actions

Unlike conventional planning domains that drive task execution, the localization domain does not perform decision-making or action selection. Instead, its actions serve as an intermediate reasoning mechanism for constructing appropriate semantic constraints. In contrast to Section 3.3, which merely validates or rejects information in a binary manner, localization actions actively update continuous state-related quantities based on the inferred semantic and geometric context. By propagating refined hypotheses and adjusted measurements to update factor values, localization actions serve as a preparatory stage that filters, structures, and enriches information, enabling the optimization backend to infer the vehicle’s state with greater robustness.

##### Semantic Candidate Inference for Localization

This stage does not directly estimate the vehicle’s final pose but instead identifies semantically valid regions that may serve as plausible locations for downstream factor construction. The inference procedure integrates the ego vehicle’s previous symbolic state with current observations to propose spatially coherent places, refines these candidates based on the vehicle’s intended behavior or high-level plan, and excludes any place whose constituent unit-level cells are occupied by obstacles. By filtering out hypotheses that violate semantic consistency or structural feasibility, this step provides the backend optimization pipeline with a compact, context-aware set of candidate locations from which meaningful factors can later be instantiated.

Listing 6 presents example PDDL actions used to infer semantically consistent candidate places before factor construction. The first action proposes new candidates based on the ego vehicle’s previous symbolic state and the topological connectivity of the semantic map. The second action filters out candidates whose unit-level cells are already occupied, ensuring compatibility with grid-map-based occupancy reasoning. These actions do not perform localization by themselves but prepare structurally valid hypotheses from which appropriate factors, such as **place-consistency factors**, **signed-distance-field (SDF) [61] factors**, and **obstacle factors** introduced in Section 4.5.1 can be instantiated reliably.

**Listing 6.** Example Actions for Semantic Candidate Inference.(:action propose_candidate_place
 :parameters (?e - ego ?prev - place ?p - place)
 :precondition (and
   (is_located_at ?e ?prev)
   (is_connected_to ?prev ?p)
   (is_reachable_to ?prev ?p)
 )
 :effect (and
   (candidate ?p)
 )
) 
(:action propose_candidate_unit_place
 :parameters (?p - place ?u - unit_place ?o - object)
 :precondition (and
   (candidate ?p)
   (is_inside_of ?u ?p)
   (is_occupied_by ?o ?u)
 )
 :effect (and
   (not (candidate ?p))
 )
)

##### Regulatory Constraint Inference: Speed, Orientation, and Restricted Zones

This step extracts regulatory constraints such as direction of travel, speed limits, and restricted or prohibited regions from the semantic HD map and aligns them with the ego vehicle’s candidate locations. Rather than estimating the vehicle’s pose directly, the objective is to prepare structured constraints that will later be converted into heading-consistency factors, speed-limit factors, and obstacle/forbidden-zone factors in the backend optimization. By filtering out candidates whose regulatory conditions violate the traffic rules encoded in the map, the system ensures that only legally and physically feasible hypotheses remain for factor construction.

The regulatory actions in Listing 7 filter out implausible place and unit-place candidates by enforcing road constraints such as heading direction, speed limits, and restricted zones.

These filtered outputs are later converted into **place-consistency factors**, **speed-limit prior factors**, and **signed distance field (SDF) factors** during backend optimization, providing stronger semantic constraints for reliable pose estimation.

**Listing 7.** Example of Actions for Regulatory Constraint Inference.(:action check_heading_regulation
 :parameters (?e - ego ?p - place ?dir - orientation)
 :precondition (and
   (candidate ?p)
   (direction ?p ?dir)
 )
 :effect (and
   (candidate ?p)
 )
)
 
(:action check_limit_regulation
 :parameters (?e - ego ?p - place ?v - velocity)
 :precondition (and
   (candidate ?p)
   (speed_limit ?p ?vmax)
   (<= ?v ?vmax)
 )
 :effect (and
   (candidate ?p)
 )
)
 
(:action check_restricted_zone
 :parameters (?p - place)
 :precondition (and
   (candidate ?p)
   (is_restricted ?p)
 )
 :effect (and
   (not (candidate ?p))
 )
)

##### Inference Valid Objects: Removing False Positives for Graph Matching

This action identifies objects that are spatially consistent with the candidate place and reliably observed by the sensor, allowing the system to filter out false positives and retain only trustworthy semantic elements for downstream graph matching.

Listing 8 identifies only those objects that are spatially valid and sensor-reliable, ensuring that false positives are eliminated before constructing semantic factors. The selected objects later serve as inputs to landmark-based constraints such as the **Landmark Factor** and **Landmark Observation Consistency Factor**, which evaluate whether the observed landmark configuration matches the HD-map structure during localization.

**Listing 8.** Example of Actions for Vaild Object.(:action check_vaild_object
 :parameters (?s - sensor ?o - object ?p - place ?)
 :precondition (and
   (candidate ?p)
   (is_measure_to ?s ?o)
   (is_inside_of ?o ?p)
   (has_object ?p)
 )
 :effect (and
   (candidate ?o)
 )
)
 
(:action check_vaild_object_relation
 :parameters (?s - sensor ?o1 - object ?o2 - object ?p - place ?)
 :precondition (and
   (candidate ?p)
   (is_measure_to ?s ?o1)
   (is_measure_to ?s ?o2)
   (is_inside_of ?o2 ?p)
   (is_near_by ?o1 ?o2)
   (has_object ?p)
 )
 :effect (and
   (candidate ?o1 ?o2)
 )
)

##### Reliability-Aware Factor Activation

This step does not determine the vehicle’s state directly but filters which sensor-generated observations are allowed to instantiate factors in the graph. By evaluating the reliability of each sensor and location, the system activates only factors from high-confidence measurements while suppressing those from uncertain or low-quality data. This selective gating not only improves semantic and numerical stability but also reduces computational load by preventing unreliable multi-sensor observations from unnecessarily increasing the factor graph size.

This action adjusts the **between-factor** weight based on the place-dependent sensor reliability value, allowing the factor graph to down-weight or up-weight observations depending on how reliable each sensor is within the corresponding semantic region (see Listing 9).

**Listing 9.** Example of Actions for for Sensor Reliability.(:action update_sensor_status
    :parameters (?s - sensor ?p - place ?r - reliability
                             ?th1 - reliability ?th2 - reliability)
    :precondition (and
          (is_reliable ?s ?p)
          (is_working ?s)
    )
    :effect (and
            (when (< ?r ?th1)
                        (not (is_reliable ?s)))
            (when (< ?r ?th2)
                        (not (is_working ?s)))
    )
)

These reasoning outcomes function as semantic constraints that directly influence the weight, structure, and validity of the factors incorporated into the localization graph. By embedding such context-aware adjustments, the estimator remains aligned with the high-level semantic interpretation of the surrounding environment. Building on these mechanisms, the next section explains how the system collects and instantiates the contextual information required for real-time localization in an on-demand and fully semantic manner.

### 3.5. Semantic Localization Problem Definition

In classical PDDL usage, a **problem** file provides an instantiation of the current world: the set of objects, their initial states, and the goal conditions that a planner must satisfy. Unlike the domain, which remains fixed, the problem is context-dependent and must reflect the environment at execution time.

For semantic localization, the problem instance is built by selectively retrieving only the information required for localization *at the current moment*, ensuring that the reasoning process reflects the vehicle’s most immediate spatial and perceptual context. Instead of preloading the full HD map or all possible semantic elements, the system delivers just the subset of relevant places, objects, and relations needed for localization at that specific timestep. This on-demand formulation minimizes computational overhead, avoids unnecessary symbolic reasoning, and maintains tight alignment between real-time localization and semantic inference.

#### 3.5.1. Semantic Problem Generation from On-Demand Database

The semantic problem instance is generated by querying the Semantic HD Map for local geometry, visible objects, spatial relations, and regulatory attributes associated with the vehicle’s estimated location, while simultaneously incorporating real-time sensing information such as detected objects, lane markings, and sensor reliability. By combining HD-map semantics with live perceptual evidence, the system constructs a problem instance that accurately reflects the vehicle’s immediate environment and sensor state, ensuring that symbolic reasoning is grounded in both map-derived knowledge and current observations.

The initial state contains the following components:

- Surrounding **candidate places and unit-places** relevant to the ego pose
- Map **connectivity, containment, and topology** (e.g., *is_connected_to*, *is_inside_of*)
- Object and lane semantics registered in each place (HDmap priors)
- Real-time sensor observations, such as detected landmarks or lane markings

By generating the problem instance dynamically, the system eliminates irrelevant regions and reduces the combinatorial space of symbolic reasoning, allowing the planner to focus only on contextually meaningful hypotheses.

#### 3.5.2. Semantic Reasoning-Driven Localization Constraints

Semantic reasoning does not directly estimate the vehicle’s pose; instead, it exposes high-level semantic information that can be injected as constraints into the localization graph. Using POPF (Partial-Order Planning Forward) [62], the reasoning engine evaluates which predicates, relations, and regulatory conditions are logically consistent with the current environment. The resulting symbolic outcomes are then converted into constraints that guide downstream geometric optimization. In this way, semantic reasoning narrows the solution space before factor-graph optimization begins, ensuring that only contextually valid hypotheses are preserved.

Moreover, once these semantic constraints are injected into the localization graph, they naturally reshape the clique structure by reinforcing only the geometrically and semantically compatible associations among places, objects, and sensor observations. Semantic factors derived from reasoning outcomes suppress inconsistent variable groupings and strengthen those that align with the scene’s contextual semantics. As a result, the optimization process converges toward hypotheses that are not only metrically plausible but also semantically coherent, yielding more stable and interpretable localization performance in complex environments.

#### 3.5.3. Semantic Reasoning-Driven Map Update

OWL-based semantic reasoning is employed to filter out implausible hypotheses by inferring logically consistent conclusions from the currently available information. Rather than processing all map elements exhaustively, only semantically relevant information is selectively queried, reducing ambiguity and preventing the propagation of invalid assumptions. Based on the refined semantic state, a PDDL-based planner determines the set of feasible actions that the robot can execute under the current conditions. The outcomes of this planning process are used to update the robot’s internal state and environment representation, and the resulting changes are reflected incrementally in the map. Through this interaction, semantic reasoning constrains the hypothesis space, while symbolic planning drives map updates in a context-aware and action-consistent manner.

## 4. Localization with Semantic Reasoning

This study proposes a multi-sensor localization framework that leverages contextual semantic understanding to achieve scalable and robust localization in complex real-world environments. To meet the requirements of autonomous driving, the system supports long-term, city-scale operation under adverse conditions, including sensor degradation and severe weather. By incorporating semantic cues beyond purely geometric measurements, including object relations and road context, the proposed approach enables robust sensor fusion and reliable localization even when geometric information becomes unreliable.

Building upon semantic information, the proposed framework first selects only the context-relevant environmental knowledge required for the current situation, forming an on-demand semantic representation. This representation is then combined with incoming sensor observations to construct a problem that reflects the current state. Based on this problem formulation, the system performs action-level planning to determine how localization should be influenced by semantic evidence, without executing physical motion. The resulting planned actions are categorized into three localization-related operations: semantic factor generation, factor suppression or reweighting, and semantic information update within the localization process.

These actions are explicitly mapped to the following three localization-relevant operations:

- **Semantic constraint generation:** semantic constraints are generated to reinforce localization hypotheses that are consistent with the current contextual and semantic state.
- **Semantic constraint suppression and reweighting:** semantic constraints that become unreliable, contradictory, or contextually invalid are selectively removed or down-weighted to prevent them from adversely affecting state estimation.
- **Semantic information update:** semantic data are updated to reflect newly inferred or validated environmental states, ensuring that subsequent reasoning remains context-consistent.

### 4.1. Dual-Loop Architecture for Real-Time and Semantic-Aware Localization

While achieving high localization accuracy is essential, SLAM and vehicle pose estimation systems must also satisfy strict real-time constraints to ensure stable operation in dynamic environments. To meet these real-time requirements and enable semantically informed localization, the proposed system adopts a dual-loop architecture comprising a real-time loop and a semantic optimization loop. This structural separation allows fast geometric processing to be executed deterministically within the real-time loop. At the same time, higher-level semantic reasoning is performed asynchronously in the optimization loop without violating latency constraints.

#### 4.1.1. Online Real-Time Localization Layer

The online layer operates under latency constraints and processes heterogeneous sensor measurements in real time. Because each sensor modality runs within an independent, fully decoupled front-end pipeline, their observations arrive with different delays and temporal characteristics. To address this asynchrony, the system employs a lightweight local factor graph that performs short-horizon temporal optimization, as is standard in graph-based estimation.

Furthermore, semantic information inferred by the offline semantic optimization layer is used to adaptively modulate sensor weights or selectively exclude unreliable observations, thereby suppressing the influence of inappropriate data and reducing computational cost. By constraining the online layer to short temporal windows and low-level geometric cues, the system maintains real-time performance while remaining robust to degraded or partially missing sensor observations.

To explicitly model increased uncertainty in semantic observations, we inflate the covariance of the corresponding semantic factors in a controlled manner within the on-demand semantic database, constrained by the spatial region where the vehicle can plausibly exist. The proposed scheme linearly increases the uncertainty up to the boundaries of the drivable road region, thereby attenuating the influence of semantic factors during optimization while preventing the emergence of physically implausible localization hypotheses.

#### 4.1.2. Offline Semantic Optimization Layer

The offline layer leverages high-level semantic information from the Semantic Modeling Framework (SMF), using semantic factors derived from object relations, scene context, HD map semantics, and symbolic-domain reasoning results. These semantic constraints enable the system to correct accumulated drift, enforce global consistency, and resolve ambiguities that purely geometric methods cannot address. Because this layer is free of real-time constraints, it can incorporate multi-session data, operate over long temporal windows, and leverage richer semantic reasoning, ultimately yielding globally consistent, semantically grounded localization estimates.

#### 4.1.3. Integrated Operation

**Figure 6:**
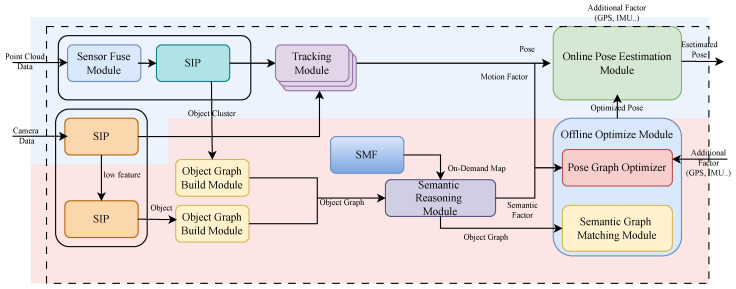
Online-offline dual-loop structure for geometric and semantic localization.

The two layers operate in a complementary manner: the online module maintains continuous real-time localization, while the offline semantic module periodically refines the trajectory and updates the semantic state. Corrections produced by the offline layer are fed back into the online system, enabling long-term drift suppression without compromising real-time responsiveness. As illustrated in Figure 6, this dual-loop interaction forms a closed feedback loop in which geometric real-time estimation and high-level semantic reasoning reinforce one another. Such an architecture allows autonomous vehicles to meet the computational requirements of real-time operation while simultaneously satisfying the semantic clarity and reasoning capability enabled by high-level contextual understanding.

### 4.2. Distributed Processing Architecture for Robust and Real-Time Operation

Traditional SLAM systems already impose significant computational demands due to dense geometric representations and real-time sensor processing. Adding a semantic reasoning layer, therefore, raises concerns regarding latency, especially in time-critical applications such as autonomous driving. To address this issue, the proposed system adopts a distributed processing architecture that separates real-time localization from semantic reasoning across multiple hardware modules.

Time-critical geometric estimation and sensor fusion are performed within the online localization loop on real-time-capable processors, while semantic reasoning and long-horizon optimization are handled asynchronously in offline modules. To account for communication delays and asynchronous updates introduced by this distributed execution, the system employs a graph-based formulation that naturally integrates delayed information without violating consistency. This design allows semantic feedback to be incorporated when available, without disrupting real-time operation.

In addition to computational efficiency, the distributed architecture improves system robustness. Because processing modules are decoupled, the failure of an individual module does not cause a complete system shutdown; instead, the remaining modules continue operating using the most recent valid estimates. This enables graceful degradation and ensures stable operation under partial hardware or communication failures. Overall, the proposed architecture integrates semantic reasoning into the localization framework while maintaining real-time performance and system reliability.

### 4.3. Background: Motion Models and Sensor Models

This section summarizes the motion and sensor models that underpin the proposed localization framework. These models support both online real-time estimation and offline semantic optimization.

#### 4.3.1. Vehicle Kinematic and Dynamic Models

**Figure 7:**
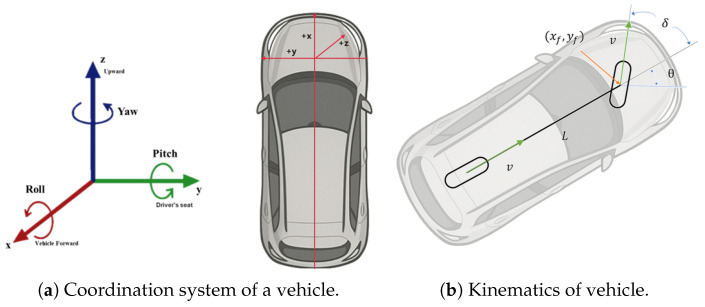
Vehicle coordinate system and kinematic model used for localization and control.

We employ a standard bicycle-model kinematic formulation to describe the vehicle motion. The state at time *k* is defined as

(7) $${\vec{x}}_{k}={\left(x_{k},\;y_{k},\;\theta_{k},\;{\dot{x}}_{k},\;{\dot{y}}_{k},\;{\dot{\theta }}_{k}\right)}^{T}$$

and the control input is modeled as

(8) $$u_{k}={\left(v_{k},\;\delta_{k}\right)}^{T},$$

where $v_{k}$ denotes the longitudinal velocity and $\delta_{k}$ is the steering angle. The continuous-time kinematic model, illustrated in Figure 7, is written as follows:

(9) $$\begin{array}{cc} {\dot{x}}_{f} & =v\cos\;(\theta +\delta )\\ {\dot{y}}_{f} & =v\sin\;(\theta +\delta )\\ \dot{\theta } & =\frac{v\sin\;(\delta )}{L} \end{array}$$

To integrate this vehicle motion model into the factor-graph framework, we impose a dynamics-consistency constraint between the pose $x_{i}$ and the corresponding velocity state $v_{i}$. The resulting residual is formulated as

(10) $$f^{\mathrm{vehicle}}\left(x_{i},v_{i}\right)={∥r_{\mathrm{vehicle}}\left(x_{i},v_{i}\right)∥}_{\Sigma }^{2},$$

where $r_{\mathrm{vehicle}}\left(\cdot \right)$ encodes the deviation between the measured or implied vehicle motion and the output of the bicycle model, and Σ represents the associated uncertainty.

These dynamic constraints act as a soft prior that encourages physically plausible motion, regularizes optimization in poorly observable situations, and suppresses unrealistic rapid changes in curvature or acceleration [59]. Moreover, the selection and activation of these constraints are adaptively guided by the robot’s semantic context described in Section 3, allowing the motion prior to be tailored to the surrounding environment.

#### 4.3.2. IMU Motion and Bias Model

The IMU provides high-rate measurements of angular velocity and linear acceleration. Each raw measurement is modeled as

(11) $$\begin{array}{cc} \tilde{\omega } & =\omega +b_{\omega }+n_{\omega }\\ \tilde{a} & =a+b_{a}+n_{a} \end{array},$$

where $b_{\omega }$ and $b_{a}$ denote slowly varying gyroscope and accelerometer biases, and $n_{\omega }$, $n_{a}$ are zero-mean Gaussian noise terms. The bias dynamics follow a random-walk model:

(12) $$\dot{b}=n_{b},$$

which captures thermal drift and long-term instability.

In the factor-graph formulation, IMU measurements are preintegrated between two keyframes to form a relative motion observation:

(13) $$z_{\mathrm{imu}}={\left(\Delta R,\;\Delta v,\;\Delta p\right)}^{T}$$

representing preintegrated rotation, velocity change, and position change. This observation is incorporated through the GTSAM IMU (preintegration) factor:

(14) $$f^{\mathrm{imu}}\left(x_{i},x_{i+1},b_{i}\right)={∥\;r_{\mathrm{imu}}\left(x_{i},x_{i+1},b_{i}\right)∥}_{\Sigma }^{2}$$

where $r_{\mathrm{imu}}\left(\cdot \right)$ is the preintegration residual, and $\Sigma_{\mathrm{imu}}$ encodes the propagated IMU noise and bias uncertainties [63].

#### 4.3.3. Camera Projection Model

The camera measurement follows the standard pinhole projection model:

(15) $$u=\pi \left(P\right)=\left[\begin{array}{c} f_{x}P_{x}/P_{z}+c_{x}\\ f_{y}P_{y}/P_{z}+c_{y} \end{array}\right]$$

where $\left(f_{x},f_{y},c_{x},c_{y}\right)$ denote the intrinsic parameters. Using this projection model, visual odometry (VO) estimates the relative motion between consecutive frames by matching keypoints and minimizing reprojection errors. The resulting observation is represented as

(16) $$z_{\mathrm{cam}}={\left(\Delta x_{\mathrm{cam}},\;\Delta y_{\mathrm{cam}},\;\Delta \theta_{\mathrm{cam}}\right)}^{T}$$

which corresponds to the incremental pose change extracted from the full SE(3) camera motion and projected onto the vehicle’s planar kinematic model. In the factor-graph formulation, this relative pose is incorporated as a between-factor:

(17) $$f^{\mathrm{cam}}\left(x_{i},x_{i+1}\right)={∥\;T_{i}^{-1}T_{i+1}-{\hat{T}}_{\mathrm{cam}}∥}_{\Sigma }^{2}$$

where ${\hat{T}}_{\mathrm{cam}}$ is the VO-derived relative transform, and $\Sigma_{\mathrm{cam}}$ is the associated covariance [7].

#### 4.3.4. LiDAR Measurement Model

LiDAR scan matching provides relative pose measurements by aligning consecutive point clouds and minimizing geometric residuals. The resulting observation is expressed as

(18) $$z_{\mathrm{lidar}}={\left(\Delta x_{\mathrm{lidar}},\;\Delta y_{\mathrm{lidar}},\;\Delta \theta_{\mathrm{lidar}}\right)}^{T}$$

with an associated covariance that reflects the uncertainty of the registration. In the factor-graph formulation, this relative motion measurement is introduced as a between-factor:

(19) $$f^{\mathrm{lidar}}\left(x_{i},x_{i+1}\right)={∥T_{i}^{-1}T_{i+1}-{\hat{T}}_{\mathrm{lidar}}∥}_{\Sigma }^{2}$$

where ${\hat{T}}_{\mathrm{lidar}}$ is the LiDAR-derived relative transform. Because this measurement constrains the change in pose rather than the absolute position, it effectively acts as a motion prior that captures short-term vehicle dynamics and complements other sensing modalities such as camera odometry and IMU integration [13,64].

#### 4.3.5. GNSS/RTK Observation Model

GNSS/RTK provides absolute global position measurements:

(20) $$z_{\mathrm{rtk}}={\left(x_{rtk},y_{rtk}\right)}^{T}$$

with associated covariance $\Sigma_{\mathrm{gps}}$. These observations are incorporated into the estimation framework as absolute priors on the vehicle’s global position. The corresponding factor is expressed as

(21) $$f^{\mathrm{gps}}\left(x_{i}\right)={∥p\left(x_{i}\right)-{\hat{p}}_{i}^{\mathrm{gps}}∥}_{\Sigma }^{2}$$

where $p(x_{i})$ denotes the position extracted from the state $x_{i}$ and ${\hat{p}}_{i}^{\mathrm{gps}}$ is the GNSS-derived position. Because GNSS/RTK measurements are expressed in a global coordinate frame, they serve as anchor points that constrain the trajectory to a consistent global reference, thereby preventing unbounded drift that typically arises in purely relative estimation systems such as visual or LiDAR odometry [63].

### 4.4. Online Real-Time Localization Layer

The online layer is responsible for generating low-latency motion estimates that can be consumed directly by the vehicle control system. Due to the distributed sensing and inference across multiple heterogeneous computing modules, asynchronous delays are unavoidable. To address this, the system adopts a short-horizon factor-graph formulation that explicitly models temporal evolution.

**Figure 8:**
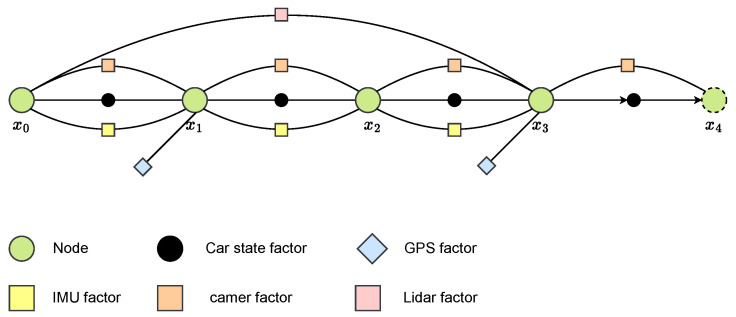
Example of the short-horizon factor-graph structure used in the online localization layer.

As illustrated in Figure 8, each incoming sensor measurement contributes a temporal or kinematic constraint between consecutive states within the short optimization window. When multiple sensors operate at different rates, the number of inter-node constraints naturally increases, and the optimizer incorporates all available velocity, orientation, and IMU-derived motion cues to refine the trajectory over the local window. This structure enables the estimator to predict the next state from the most recently received velocity or inertial input, even when other sensor measurements arrive with a delay, allowing the vehicle to maintain stable real-time motion estimation.

Additionally, by leveraging the semantic information provided by the Offline Semantic Optimization Layer in Section 4.5, the system can dynamically adjust sensor weights. This allows for the exclusion or attenuation of unreliable sensor measurements or distorted data, resulting in more accurate and stable localization estimates. This approach reflects the differences in sensor reliability, ensuring that uncertain information does not unduly influence the optimization process.

The state maintained in this layer focuses on the primary vehicle dynamics and is defined as

(22) $$x_{k}=\left(T_{k},\;v_{k},\;b_{k}^{\mathrm{imu}}\right)$$

where $T_{k}\in SE\left(2\right)$ denotes the vehicle pose, $v_{k}$ the planar velocity, and $b_{k}^{\mathrm{imu}}$ the gyroscope and accelerometer bias. This state vector is intentionally restricted to the essential motion variables to satisfy the strict real-time constraints of the online layer.

For estimation, the online layer maintains a compact factor graph over a sliding temporal window. Let $X_{0:N}$ denote the active state variables and F the set of factors constructed from all available sensor measurements. The short-horizon estimation problem is formulated as

(23) $${\hat{X}}_{0:N}=\arg\;\min_{X_{0:N}}\sum_{f\in F}∥r_{f}\left(X_{0:N}\right){∥}_{\Sigma_{f}}^{2},$$

where $r_{f}$ is the residual of factor *f* and $\Sigma_{f}$ its covariance. The optimization is solved using the Levenberg–Marquardt algorithm [65], which provides rapid and stable convergence even during rapid vehicle motion.

To meet real-time requirements, the local graph is maintained over a short horizon, and sensor measurement influence is adaptively adjusted using the semantic reasoning described in Section 4.5.1. Unreliable measurements are re-scaled or removed to prevent error propagation during estimation, enabling effective fusion of heterogeneous sensor data while preserving real-time performance. Moreover, asynchronous delays are handled by managing measurements within a temporal graph, ensuring consistent alignment while preserving real-time responsiveness for autonomous driving. Moreover, asynchronous delays are addressed by managing measurements within a temporal graph, enabling consistent alignment and improving estimation reliability.

### 4.5. Offline Semantic Optimization Layer

The offline layer performs large-horizon optimization to globally refine the trajectory and calibration states produced by the online estimator. By integrating geometric residuals, semantic HD-map consistency, and object-place relations over extended temporal windows, it corrects drift and resolves ambiguities that cannot be handled under online constraints. The refined states and reliability information computed offline are then propagated to Section 4.5, where they are used to update semantic priors and improve the robustness of subsequent online estimation.

#### 4.5.1. Semantic Constraint for Global Optimization

Based on the semantic reasoning defined in the Semantic Modeling Framework, the offline layer incorporates these semantic relations into the numerical optimization process by formulating them as factor-level residuals. Whereas the online estimator focuses on short-horizon motion cues, the offline module utilizes place semantics, object relations, regulatory attributes, and landmark configurations extracted from the SMF to enforce long-range contextual consistency. Through these semantic factors, the optimization is constrained to trajectories and calibration states that are not only geometrically plausible but also semantically aligned with the HDmap-based environmental structure.

##### Place-Consistency Factor

The place-consistency factor ensures that the estimated vehicle pose remains geometrically and semantically consistent with the corresponding place in the HD map. For a given semantic place *p*, the residual is formulated as

(24) $$f^{\mathrm{place}}\left(x_{i},p\right)=\;∥r_{\mathrm{place}}\left(x_{i},p\right){∥}_{\Sigma }^{2},$$

where the consistency residual is defined by

(25) $$r_{\mathrm{place}}\left(x_{i},p\right)=\left[\begin{array}{c} d_{\mathrm{geom}}\left(x_{i},p\right)\\ d_{\mathrm{orient}}\left(x_{i},p\right) \end{array}\right]$$

Here, $d_{\mathrm{geom}}$ denotes the geometric distance from the vehicle position to the lane or region boundary associated with the place, and $d_{\mathrm{orient}}$ is the orientation deviation between the vehicle heading and the canonical direction of that semantic region. The covariance $\Sigma_{\mathrm{place}}$ encodes the confidence of the semantic place definition.

##### Signed-Distance-Field (SDF) Factor

The SDF factor penalizes trajectories that violate roadway boundaries, safety regions, or obstacle margins encoded in the signed-distance field ϕ(·). The factor is expressed as

(26) $$f^{\mathrm{sdf}}\left(x_{i}\right)=\;{∥r_{\mathrm{sdf}}\left(x_{i}\right)∥}_{\Sigma }^{2}$$

with the residual defined as

(27) $$r_{\mathrm{sdf}}\left(x_{i}\right)=\phi \left(p(x_{i})\right)$$

where ϕ(·) returns the signed distance between the vehicle position and the nearest boundary or obstacle. Positive values indicate free space, values near zero represent boundary contact, and negative values indicate that the estimated pose lies within an invalid or occupied region. The weighting matrix Σ controls the strength of the penalty, typically assigning higher penalties to negative distances.

##### Speed-Limit Prior Factor

The speed-limit prior factor penalizes velocity estimates that exceed the maximum permissible speed defined for the corresponding map region. Let $v_{i}$ denote the estimated longitudinal velocity and $v_{\max}\left(p\right)$ the speed limit associated with place *p*. The residual is defined as

(28) $$f^{\mathrm{spd}}\left(x_{i},p\right)={∥r_{\mathrm{spd}}\left(x_{i},p\right)∥}_{\Sigma }^{2}$$

(29) $$r_{\mathrm{spd}}\left(x_{i},p\right)=\max\left(0,\;v_{i}-v_{\max}\left(p\right)\right)$$

##### Landmark Factor

The landmark factor enforces geometric consistency between the ego pose and a mapped landmark by comparing the actual landmark measurement with the expected observation predicted from the HD-map.

(30) $$r_{k,i}^{\mathrm{lm}}={\hat{z}}_{k,i}^{\mathrm{lm}}-h\;\left(T_{k},p_{i}^{\mathrm{map}}\right)$$

This factor penalizes discrepancies between measured and predicted landmark observations, anchoring the trajectory to semantically stable map features. Here, the observation model $h(T_{k},p_{i}^{\mathrm{map}})$ predicts how the mapped landmark should appear in the sensor frame given the ego pose and sensor characteristics.

##### Landmark Observation Consistency Factor

The landmark observation consistency factor assesses whether the relative configurations of multiple observed landmarks match the relational structure encoded in the semantic HD map.

(31) $$r_{k,(i,j)}^{\mathrm{rel}\text{-}\mathrm{lm}}=\left({\hat{z}}_{k,i}^{\mathrm{lm}}-{\hat{z}}_{k,j}^{\mathrm{lm}}\right)-\left(p_{i}^{\mathrm{map}}-p_{j}^{\mathrm{map}}\right)$$

By constraining relative landmark geometry, this factor improves robustness in repetitive or ambiguous environments and ensures semantic consistency across frames.

##### Landmark Visibility Factor

The landmark visibility factor imposes a weak semantic constraint based on whether a landmark should be visible from the current pose and sensor field of view.

(32) $$r_{k,i}^{\mathrm{vis}}=I\;\left({\hat{v}}_{k,i}\right)-I\;\left(v_{k,i}^{\mathrm{map}}\left(T_{k}\right)\right)$$

This residual penalizes cases where a landmark is expected to be visible but is not observed (or vice versa), providing a geometry-light constraint ideal for low-accuracy contexts; here, the indicator function I(·) returns 1 when the visibility condition is satisfied and 0 otherwise, allowing the factor to capture semantic inconsistencies without relying on precise geometry.

#### 4.5.2. Multilayer Object Graph Matching

In the proposed framework, object-level information extracted from multiple sensors is fused by explicitly accounting for sensor-specific characteristics. Camera observations provide rich semantic labels but often suffer from missed detections, whereas LiDAR measurements offer accurate geometry with higher false positives and limited semantic labeling. Leveraging known camera-LiDAR extrinsic calibration and HD-map anchors, object hypotheses from heterogeneous sensors are associated and refined in a unified object-centric representation, where complementary strengths across modalities are systematically exploited. Crucially, only object information that is deemed reliable after this semantic and cross-sensor reasoning process is injected into the localization graph. Rather than densely populating the pose graph with all available measurements, the system selectively introduces geometrically and semantically consistent object constraints, ensuring that the estimation remains robust to sensor noise, misdetections, and spurious observations. This design enables the localization backend to operate on a compact and reliable factor graph while still benefiting from rich multi-sensor semantic context, constituting a key contribution of the proposed framework.

Such vehicle-object and object-object relational representations can be naturally extended to cooperative perception by defining inter-vehicle relationships through vehicle-to-vehicle (V2V) communication.

##### Object Association Factor

Given two nodes $o_{i}$ and $o_{j}$ that represent the same physical object but originate from different layers (e.g., camera, LiDAR, or HD-map), the association factor enforces that both nodes converge to a common 3D location. This factor is modeled as

(33) $$f_{\mathrm{assoc}}\left(o_{i},o_{j}\right)={∥\;p_{o_{i}}-p_{o_{j}}\;∥}_{\Sigma }^{2},$$

where $p_{o_{i}}$ and $p_{o_{j}}$ denote the object positions in the global reference frame. $\Sigma_{\mathrm{assoc}}$ represents the expected uncertainty of cross-layer matching. This factor effectively binds multimodal object detections into a unified semantic representation.

##### Object Consistency Factor

For repeated observations of the same object by a single sensor across multiple poses, a temporal *Object Consistency Factor* is introduced. It constrains the transformed object positions to remain consistent when expressed in different vehicle poses:

(34) $$f_{\mathrm{cons}}\left(x_{k},x_{k+1},o\right)={∥\;T{\left(x_{k}\right)}^{-1}p_{o}^{k}-T{\left(x_{k+1}\right)}^{-1}p_{o}^{k+1}\;∥}_{\Sigma }^{2}$$

where T(·) is the SE(3) transformation of each pose node. This factor stabilizes object estimation over time and mitigates noise or partial detections.

##### Pose Graph Constraints

In the pose graph layer, standard relative-motion constraints are applied between consecutive states:

(35) $$f_{\mathrm{between}}\left(x_{k},x_{k+1}\right)={∥\;r(x_{k},x_{k+1})\;∥}_{\Sigma }^{2}$$

where r(·) represents the relative-pose residual. These constraints maintain smooth vehicle motion and propagate information to all connected object nodes.

##### Unified Multilayer Graph Optimization

The full multilayer factor graph, including pose constraints, object association factors, and temporal consistency factors, is optimized jointly. The global estimation problem is expressed as

(36) $$\hat{X}=\arg\;\min_{X}\left(\sum f_{\mathrm{between}}+\sum f_{\mathrm{assoc}}+\sum f_{\mathrm{cons}}\right)$$

where X includes all pose and object variables. By minimizing the joint residuals, the system aligns sensor-derived objects with HD-map landmarks while simultaneously refining the vehicle trajectory. This approach enables robust cross-sensor object matching and improves localization performance even when only a few objects are visible [66].

#### 4.5.3. Semantic Global Optimization

Global optimization integrates semantic consistency into global pose estimation by encoding semantic and contextual information as factors rather than additional state variables, enabling robust and scalable inference. Place- and object-level semantics provide contextual grounding for factor construction. At the same time, semantic relations, sensor observations, and kinematic constraints are expressed as pose-to-pose factors that reference an on-demand semantic database. This formulation avoids unnecessary expansion of the state space while ensuring consistency with map-aligned regions, object stability, and environmental semantics.

Computationally expensive geometric constraints, such as signed distance field evaluations, are parameterized at the place level and applied to pose variables through place-conditioned factors. By localizing high-cost computations at the region level and selectively activating object-related constraints only when multilayer consistency conditions are satisfied, the framework suppresses unreliable landmarks. It improves computational efficiency without sacrificing geometric fidelity.

For efficient inference, an incremental smoothing framework [66] is employed, allowing new factors to be integrated without re-optimizing the entire graph. Semantic coherence emerges naturally from factor connectivity, as constraints on places, objects, and vehicle motion induce structured dependencies among pose variables. This results in semantically and spatially meaningful cliques within the Bayes tree, enabling stable and efficient incremental updates grounded in the environment [36,67].

##### Graph Linearization

At each update step, nonlinear factors are linearized around the current estimate. Let X denote the full set of variables. Each factor $f_{i}\left(X\right)$ is approximated using its first-order Taylor expansion,

(37) $$r_{i}\left(X+\delta X\right)\approx r_{i}\left(X\right)+J_{i}\;\delta X,$$

where $r_{i}$ is the residual and $J_{i}$ is the Jacobian matrix. Stacking all factors yields the normal-equation form:

(38) $$\left(\sum_{i}J_{i}^{⊤}\Sigma_{i}^{-1}J_{i}\right)\delta X=-\sum_{i}J_{i}^{⊤}\Sigma_{i}^{-1}r_{i}.$$

##### Graph Optimization

The unified estimation problem across place, object, and pose nodes is then expressed as the global residual minimization:

(39) $$\hat{X}=\arg\;\min_{X}\sum_{i}{∥r_{i}\left(X\right)∥}_{\Sigma_{i}}^{2},$$

with incremental updates applied as new constraints arrive. This formulation ensures that semantic factors, geometric consistency, and dynamic constraints contribute jointly to the final state estimate.

### 4.6. Expansion to Cooperative Perception

The semantic factors introduced above naturally extend to cooperative multi-vehicle perception, since the formulation relies not on a single vehicle’s measurements but on relational constraints among objects, and ego-poses over time. When multiple vehicles share their locally constructed object graphs, each graph contributes additional inter-object and inter-pose constraints that enrich the global semantic structure. Because these factors encode relationships such as relative geometry, temporal consistency, and landmark visibility rather than raw sensor values, they can be fused across vehicles without requiring strict sensor-level synchronization.

**Figure 9:**
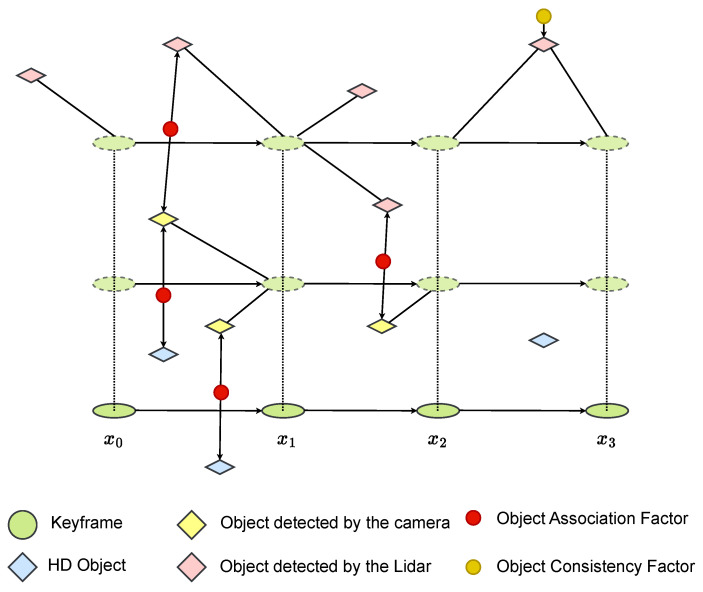
Multilayer object-pose graph structure used for object association and temporal consistency.

When two vehicles exchange their object detections or local map segments, the shared object nodes can be aligned using the previously defined *Object Association Factor*. In addition, objects repeatedly observed by each vehicle are temporally refined by the *Object Consistency Factor*. Through these factors, object-level semantics serve as a reference structure that multi-vehicles can use to align their trajectories and improve localization robustness, as shown in Figure 9.

To integrate independently estimated vehicle poses from different platforms, an inter-vehicle relational factor is introduced. Let $x_{i}^{A}$ denote the pose of vehicle A at time *i*, and $x_{j}^{B}$ denote the pose of vehicle B at time *j*. Whenever a relative spatial constraint between the two vehicles becomes available, either through direct sensing, V2V communication, or inferred alignment through shared objects, the following inter-vehicle factor is applied:

(40) $$f_{\mathrm{veh}}\left(x_{i}^{A},x_{j}^{B}\right)={∥\;r_{\mathrm{veh}}\left(x_{i}^{A},x_{j}^{B}\right)-{\hat{z}}_{\mathrm{veh}}\;∥}_{\Sigma }^{2}$$

where $r_{\mathrm{veh}}$ represents the relative-pose residual derived from the two vehicle states, ${\hat{z}}_{\mathrm{veh}}$ is the observed or communicated relative measurement, and $\Sigma_{\mathrm{veh}}$ denotes the covariance capturing the uncertainty of inter-vehicle estimation.

By incorporating the inter-vehicle factor alongside object association and object consistency factors, the multilayer graph can form a cooperative structure in which multiple vehicles jointly contribute to a unified semantic-geometric representation of the environment. This formulation is expected to facilitate the alignment of local trajectories and improve robustness in regions where individual vehicles experience limited visibility or sparse features. Ultimately, such a cooperative multilayer graph may enable heterogeneous sensors across multiple platforms to converge on a shared HDmap and object layout, supporting more reliable joint localization and shared situational awareness.

## 5. Experiment

This section presents a comprehensive experimental evaluation of the proposed semantic localization framework. The experiments are designed to validate the effectiveness, robustness, and practical applicability of the proposed approach under diverse operational conditions. Various scenarios and sensor configurations are considered to systematically analyze localization accuracy, real-time feasibility, and scalability in realistic driving environments.

### 5.1. Experimental Setup

**Figure 10:**
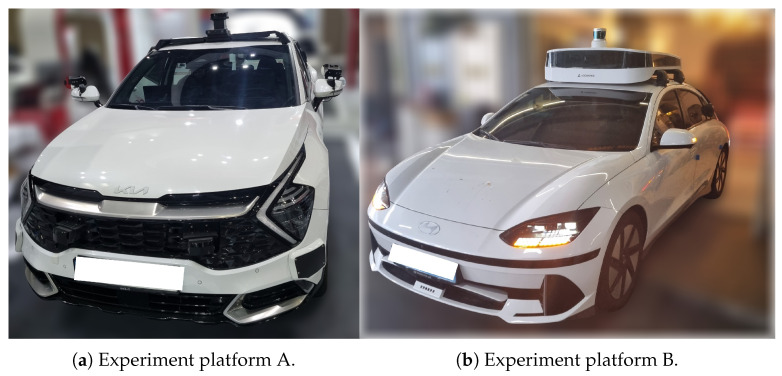
Experimental multi-sensor vehicle for adverse-condition simulation.

To evaluate the performance of the proposed localization system under diverse operational conditions, six representative experimental scenarios were selected. To closely emulate real-world driving environments, each scenario was configured using different sensor combinations, and experiments were conducted on two distinct vehicle platforms (Figure 10) to account for variations in vehicle configurations and sensor layouts. This experimental setup enables a systematic evaluation of localization performance and robustness under heterogeneous and realistic sensing conditions.

**Figure 11:**
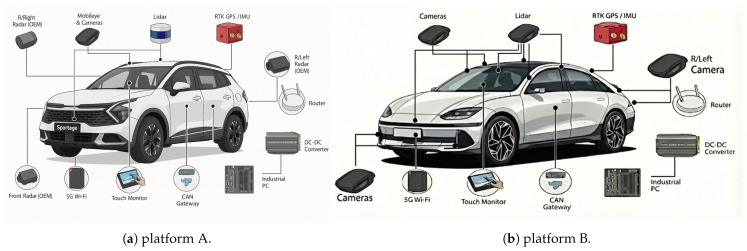
Overview of the sensor configuration of the experimental vehicle.

**Figure 12:**
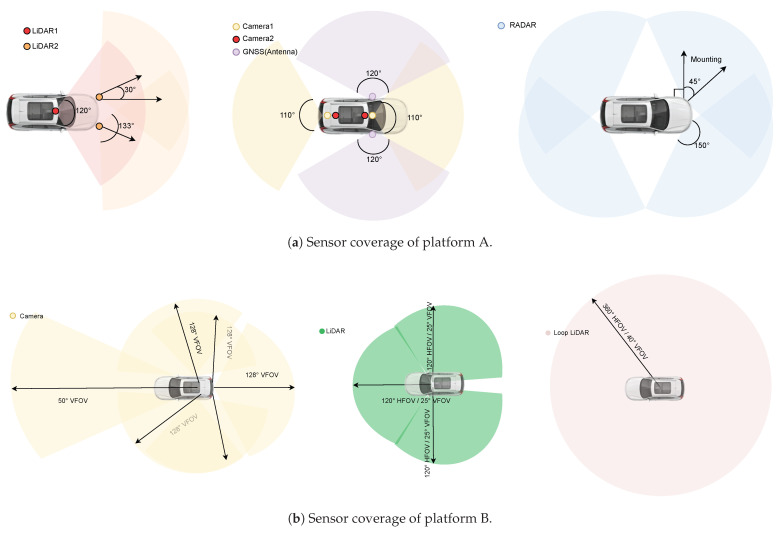
Sensor coverage of the experimental vehicle.

To ensure practical relevance to real-world autonomous driving systems, experiments were conducted using two vehicle platforms equipped with a variety of sensors commonly used in autonomous driving, including GNSS, cameras, LiDAR, and radar. Although the two platforms differ in sensor configurations and layouts, both are designed to support high-precision localization and robust perception in complex driving environments. An overview of the experimental vehicle platforms and sensor configurations is illustrated in Figure 11, while the corresponding sensor coverage characteristics are presented in Figure 12. Detailed sensor configurations for each platform are summarized in Table 8.

In addition to comprehensively evaluating the proposed localization system under a wide range of realistic operational conditions, six representative experimental scenarios were designed and systematically evaluated. Rather than relying on fixed sensor counts, each scenario uses different combinations of available sensing modalities, allowing the experimental setup to explicitly reflect heterogeneous, dynamically changing sensing conditions encountered in real-world environments. This design enables a thorough assessment of both localization accuracy and robustness when individual sensors are degraded, unavailable, or selectively utilized. The six experimental scenarios are summarized as follows:

- **Scenario 1: Nominal driving**: no adverse driving conditions.
- **Scenario 2: GPS interference**: presence of intermittent or spurious GPS signals.
- **Scenario 3: GPS disruption**: partial signal loss due to urban canyons or overpasses.
- **Scenario 4: GPS shadowing**: complete loss of GPS signals.
- **Scenario 5: LiDAR failure**: LiDAR sensor data becomes unavailable.
- **Scenario 6: Camera failure**: camera sensor data becomes unavailable.

**Figure 13:**
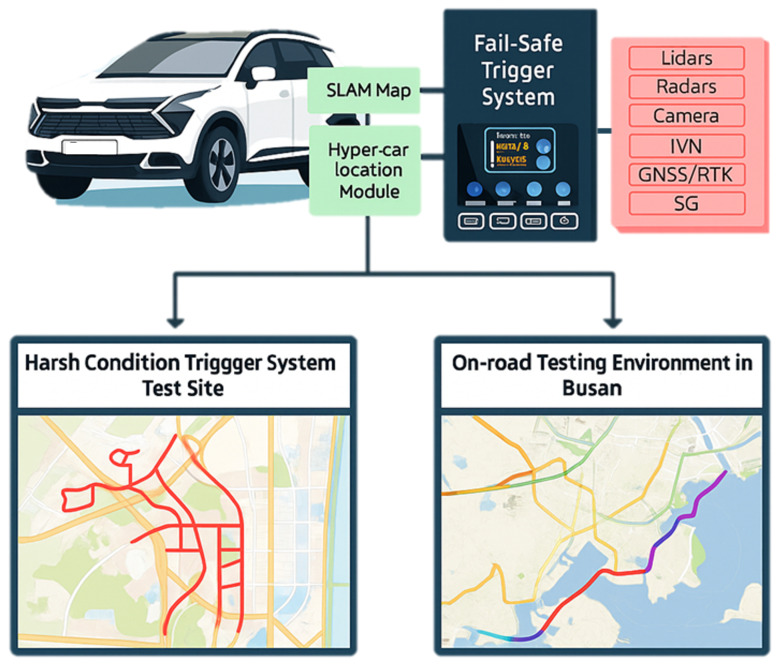
System architecture designed to simulate all six experimental scenarios.

To realize all six experimental scenarios defined in the study, the system was carefully configured as illustrated in Figure 13. The proposed setup integrates multiple sensing modalities, including GNSS for GPS-based scenarios, cameras, and LiDARs, along with dedicated control modules that enable sensor deactivation or degradation to emulate failure conditions. Each component was designed for modularity and fault-injection capabilities, enabling repeatable, scenario-specific evaluations in both virtual and real-world environments. This architecture supports flexible switching between nominal and fault conditions, enabling rapid transitions across different test modes.

**Figure 14:**
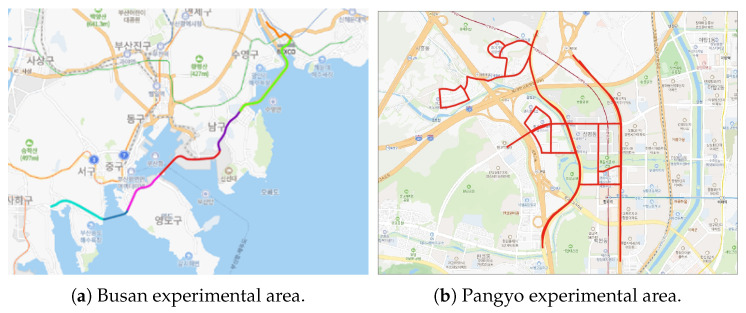
Experimental area. Busan (**a**) and Pangyo (**b**). The red region indicates the actual experimental area.

To specifically investigate GPS degradation and disturbance conditions, two representative test locations were selected to encompass a sequence of challenging environments for satellite navigation. The chosen routes include tunnels and underpasses that cause complete GPS outages, and coastal bridges where severe multipath reflections occur. Notably, the scenarios were designed to evaluate system robustness in soundproof wall segments with overhead coverage and double-deck bridge structures (lower and upper levels), which induce continuous signal weakening and Non-Line-of-Sight (NLOS) interference. By conducting experiments in these ’Urban Canyon’ and ’Signal-Denied’ successions, the proposed localization system was evaluated under realistic and extreme GPS-weak conditions [68]. Figure 14 illustrates the actual regions used for experimentation.

### 5.2. Localization Accuracy and Robustness

This section evaluates the validity of the proposed localization system through four experimental studies. The experiments are conducted sequentially to analyze performance under adverse scenarios, compare the proposed method with conventional approaches, assess feasibility for real-world vehicle deployment, and examine extensibility through cooperative perception scenarios.

#### 5.2.1. Performance Evaluation Under Environmental Variations and Sensor Failures

To evaluate the localization performance of the proposed method, we conducted a series of scenario-based experiments under varying environmental and sensor failure conditions.

To better interpret the results in Table 9, we briefly describe each performance metric [69] used for evaluating localization accuracy and robustness as follows:

- **Longitude Error (lon, m)**: The absolute localization error along the east–west direction, measured as the positional deviation between the estimated position and the ground truth.
- **Latitude Error (lat, m)**: The absolute localization error along the north–south direction, measured as the positional deviation between the estimated position and the ground truth.
- **Heading Error (rad)**: The absolute heading error, expressed in radians, represents the angular deviation between the estimated vehicle heading and the ground-truth heading.
- **Integrity Risk (PHMI/hr)**: The probability of hazardously misleading information (HMI) occurring when the true positioning error exceeds the allowable error while remaining undetected by the estimated error, expressed per hour.
- **Availability (%)**: The percentage of localization outputs that satisfy both accuracy and integrity requirements, defined as cases where the true and estimated positioning errors remain within the allowable accuracy bound, and the localization decision is provided within 100 ms.

In real-world driving environments, obtaining precise ground truth is inherently challenging. Therefore, in this study, RTK-GNSS measurements were used as ground truth only when their reliability was sufficiently ensured. In contrast, for segments where RTK signals were degraded or unavailable, a semi-ground truth was generated by combining vehicle motion constraints, including traveled distance, time, and velocity, with HD map information. During these segments, the vehicle followed a predefined route and was assumed to travel near the center of the lane. Furthermore, all experiments were conducted under normal traffic flow, and scenarios involving traffic congestion, abrupt braking, lane departure, or traffic regulation violations were explicitly excluded.

Integrity Risk is formally defined as the probability of HMI events occurring during the observation period:

(41) $$\mathrm{Integrity}\;\mathrm{Violation}\;\mathrm{Rate}=\frac{1}{N}\sum_{i=1}^{N}I_{\mathrm{HMI}}\left(i\right)$$

where $I_{\mathrm{HMI}}\left(i\right)$ is an indicator function defined as

(42) $$I_{\mathrm{HMI}}\left(i\right)=\left\{\begin{array}{cc} 1, & \mathrm{if}\;{\hat{\sigma }}_{i}<\epsilon <e_{i}\\ 0, & \mathrm{otherwise} \end{array}\right.$$

Here:

- ${\hat{\sigma }}_{i}$: predicted positioning error (system internal estimate) at time *i*
- ϵ: allowable error bound defined by annual integrity requirements
- $e_{i}$: actual positioning error at time *i*

The system satisfies the integrity requirement if the HMI occurrence rate remains below the yearly integrity threshold $\eta_{\mathrm{target}}$:

(43) $$\frac{1}{N}\sum_{i=1}^{N}I_{\mathrm{HMI}}\left(i\right)<\eta_{\mathrm{target}}$$

Availability is defined as the proportion of time during which the ego vehicle’s position data satisfies both accuracy and integrity requirements, among the entire observation period, and also meets a temporal trustworthiness constraint.

(44) $$\mathrm{Availability}=\frac{\sum_{i=1}^{N}I_{\mathrm{int}}\left(i\right)\cdot I_{\mathrm{acc}}\left(i\right)\cdot w_{t}\left(i\right)}{\sum_{i=1}^{N}w_{t}\left(i\right)}$$

where:

- $I_{\mathrm{int}}\left(i\right)$: Integrity indicator (1 if the position error at time *i* is within the defined bound; otherwise 0).
- $I_{\mathrm{acc}}\left(i\right)$: Accuracy indicator (1 if the ego-position meets the accuracy requirement; otherwise 0).
- $w_{t}\left(i\right)$: Temporal weight that decays over time to reflect recency (higher for recent data, lower for old data).

For the availability evaluation, temporal weights are computed using exponential decay, allowing a tolerance of up to 10 ms:

(45) $$w_{t}\left(i\right)=\lambda^{-(t_{\mathrm{now}}-t_{i})}$$

where λ is a decay factor slightly greater than 1, typically λ ≈ 1.005 to permit minimal degradation within 100 ms.

**Figure 15:**
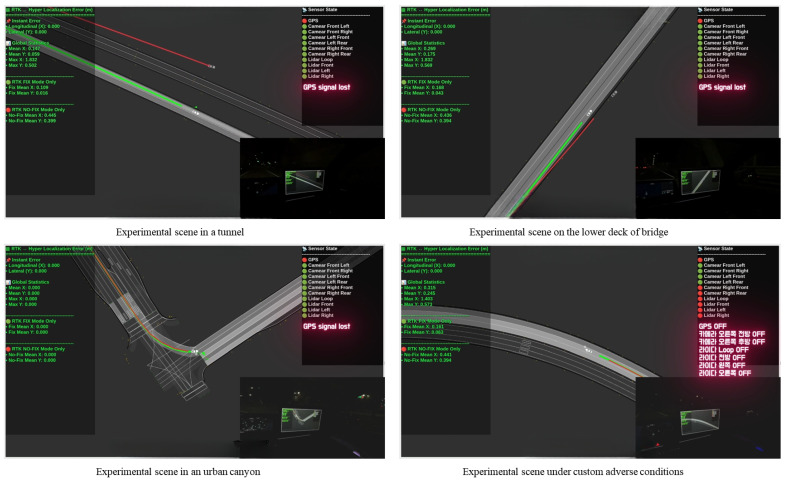
Experimental scenes under various adverse conditions.

Figure 15 illustrates real-world scenes captured during the experiments. To ensure a clear evaluation, the results are presented at two distinct levels. Table 9 summarizes the localization accuracy and robustness for each of the six representative scenarios. This categorization enables a detailed analysis of performance under specific conditions, such as tunnels or urban canyons. In contrast, Table 10 presents an aggregated view of localization performance across five full-scale trials, covering all scenarios along a continuous driving route.

The proposed localization system demonstrates sub-meter-level positioning accuracy and stable heading estimation across all evaluated scenarios. The experimental results indicate that reliable localization performance is maintained even under degraded GNSS conditions and partial sensor failures, with only limited performance variation across different environmental conditions. The consistent behavior observed across S1–S6 scenarios suggests that the localization pipeline is resilient to both GNSS-related disturbances and sensor-level disruptions, likely supported by the integration of multi-layer semantic features and sensor fusion redundancy. Overall, these findings suggest that the proposed method delivers stable, dependable localization performance, a necessary component for autonomous driving in real-world environments.

#### 5.2.2. Comparison with Other Localization Methods

To further assess the effectiveness of the proposed hierarchical semantic localization framework, we conducted a comparative evaluation against several representative localization baselines widely used in autonomous driving applications. The comparison focuses on the system’s ability to maintain localization accuracy in GPS-degraded environments, specifically in GPS-shadowed regions where RTK signals temporarily become unavailable.

The evaluated baseline methods include filtering-based estimators (EKF, UKF), LiDAR-centric SLAM frameworks (LeGO-LOAM, HDL Graph SLAM, G-ICP), and visual SLAM methods (OpenVSLAM with OpenVINO acceleration). These methods collectively represent diverse paradigms in localization probabilistic filtering, LiDAR-based scan matching, and vision-based keyframe optimization, allowing a broad comparison against the proposed multi-layer semantic approach. Table 11 presents the empty template that summarizes the comparative evaluation.

The experimental results demonstrate that, unlike conventional feature-based mapping methods that operate without explicit landmark structures, the proposed semantic multi-layer approach exhibits significantly reduced error. This improvement arises from integrating stable object- and place-level constraints, which effectively limit accumulated pose errors even under degraded sensing conditions.

#### 5.2.3. Cooperative Perception Extension Experiment

To evaluate the scalability of the proposed multilayer semantic localization framework toward cooperative perception, we conducted an additional experiment using two independently recorded vehicle datasets. Due to practical constraints, simultaneous operation of two physical vehicles along the same route was not feasible; therefore, two driving sequences were collected along routes that were similar yet slightly different within the same HD-map-enabled region.

In this experiment, RTK-GNSS measurements were intentionally not used, even in regions where RTK coverage would typically be available, and inter-vehicle information sharing was assumed to occur offline rather than in real time. Each vehicle was treated as an independent agent that shared only limited information with the other agent, namely its estimated pose trajectory and the relative distances between the agent and the recognized objects.

By comparing cases with and without such information sharing, we evaluate whether exchanging object-level semantic constraints between vehicles can reduce drift or improve positioning accuracy under GNSS-degraded conditions. Although this setup does not represent a fully synchronized, real-time, multi-vehicle system, it provides initial validation of cooperative semantic localization. It suggests that multi-agent semantic fusion can enhance localization robustness. The comparative results are summarized in Table 12.

### 5.3. Validation of SMF Effectiveness

This section presents experiments that validate the effectiveness of the proposed semantic modeling framework and the key factors it infers from the semantic information. The experiments demonstrate that semantic information not only enriches scene representation but also plays a crucial role in improving constraint reliability, optimization stability, and drift resistance across various conditions, including sensor degradation, outlier contamination, and multimodal inconsistency.

#### 5.3.1. Semantic Environmental Modeling for Experiments

In this section, we present two statistical tables that summarize the semantic environmental models constructed and used in the experiments. Table 13 and Table 14 provide an overview of the semantic object categories and their corresponding counts in each experimental environment. These tables are intended to illustrate the scale, diversity, and composition of the semantic information available in the constructed environments, rather than to report performance outcomes directly. By presenting these statistics, we clarify the experimental context in which the proposed localization framework was evaluated.

#### 5.3.2. Impact of Semantic Rules on Performance

This experiment was conducted to evaluate whether the proposed semantic factors can improve localization performance beyond that of conventional approaches. When semantic rules were applied, both longitudinal and lateral errors were reduced, and the stability of the estimated trajectory was improved, indicating consistent performance gains. These results suggest that semantic information derived from the SMF functions as an additional supervisory layer, enhancing robustness even in real-world GNSS-degraded environments.

By leveraging not only raw sensor measurements but also implicitly inferred information derived from semantic rules and environmental context, the proposed framework performs reliability-aware evaluation of sensor observations. Furthermore, a sensor reliability–based fusion strategy selectively excludes unreliable sensor measurements, effectively mitigating the impact of erroneous observations on the estimation process. This rule-based mechanism improves estimation stability without requiring modifications to the underlying sensor models, highlighting its practicality for deployment in real-world systems.

Importantly, the selective registration of semantic constraints does not introduce erroneous convergence or estimation bias; instead, it stabilizes the optimization process by enforcing only reliable and context-consistent constraints. Overall, the experimental results suggest that semantic factors provide a safe and effective means to improve the reliability and accuracy of localization systems.

**Figure 16:**
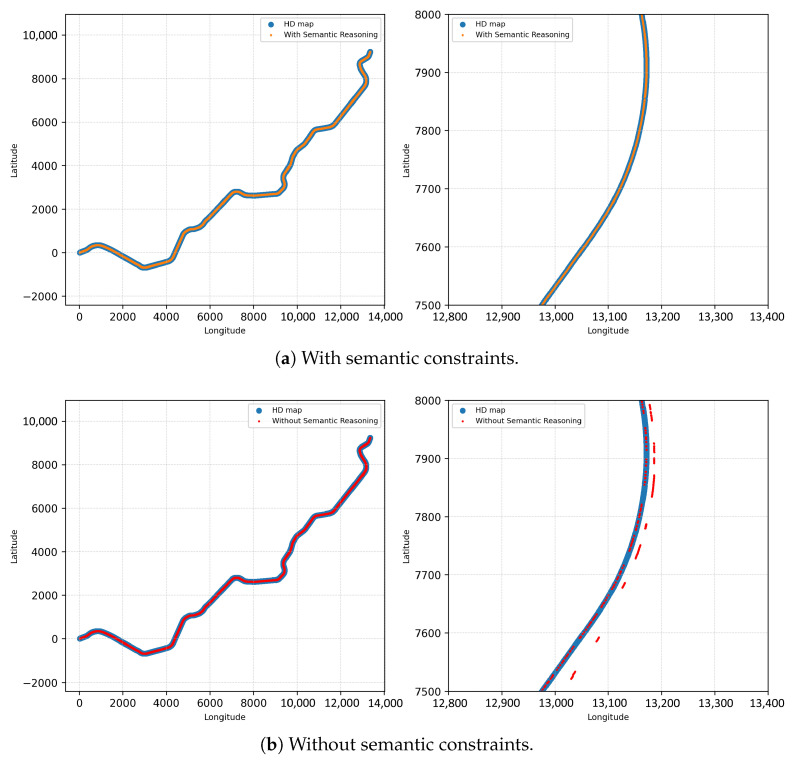
Trajectory comparison with semantic (**a**) and without semantic (**b**).

The experimental results show that leveraging semantic information enables the system to produce more stable and reliable localization estimates, particularly when sensor data is degraded or when operating in GPS-weak or shadowed environments. By incorporating semantic cues, the framework mitigates uncertainties arising from adverse sensing conditions and maintains coherent trajectory estimation, even when conventional methods struggle. In particular, as illustrated in Figure 16, the incorporation of semantic constraints significantly reduces optimization instability and divergence caused by conflicting or antagonistic constraints, which frequently lead to failure in purely geometric formulations. As summarized in Table 15, both longitudinal and lateral localization errors decrease significantly, confirming the effectiveness of the proposed semantic integration.

In addition to improving estimation accuracy and robustness, incorporating semantic information also leads to tangible computational benefits. The proposed framework builds on incremental smoothing and mapping, where optimization complexity is strongly influenced by the number of active factors and the relinearization frequency. By leveraging semantic rules to identify and reject contextually invalid or unreliable measurements, a significant portion of outlier-induced factors is filtered out before being registered in the factor graph. This reduction not only decreases the total number of factors involved in optimization but also limits unnecessary relinearization events triggered by inconsistent constraints, thereby reducing overall computational load.

Furthermore, as introduced in Section 4.2, the proposed Distributed Processing Architecture decouples semantic constraint evaluation from global graph optimization. The computation required for semantic-based factor pruning is handled independently from the core optimization process, preventing additional burden on the optimizer. As a result, semantic reasoning improves optimization efficiency rather than adding overhead.

To ensure a fair and comprehensive evaluation of computational performance, all experiments were conducted by optimizing over the entire factor graph without employing a fixed-lag smoothing strategy, thereby including all accumulated nodes and factors in the optimization process. This effect is quantitatively validated in Table 16, where the semantic-aware (Robust) mode consistently achieves lower average optimization time, dramatically fewer relinearization events, and substantially reduced total runtime compared to the standard formulation, even as the number of nodes and factors increases. These results confirm that semantic-guided factor selection improves scalability while preserving estimation stability.

To isolate the impact of semantic reasoning from generic sensor fusion effects, we perform an ablation study by selectively disabling individual semantic constraints introduced in Section 4.5.1. Each configuration removes exactly one semantic factor while keeping the remaining optimization framework unchanged. The quantitative results are summarized in Table 17.

With all semantic constraints enabled, the proposed semantic-aware optimization achieves the lowest localization errors, the best integrity, and the highest availability among all configurations. This result serves as the reference performance for evaluating the contribution of individual semantic factors.

The most significant degradation occurs when the Landmark Factor is removed. In this case, the optimization loses its ability to anchor the estimated trajectory to semantically stable map entities, resulting in pronounced degradation in accuracy, integrity, and solution availability. This behavior indicates that explicit landmark-based semantic anchoring plays a central role in enforcing global consistency and robustness within the proposed optimization framework.

Disabling the Place-Consistency and Signed-Distance-Field constraints also leads to notable performance degradation, particularly in lateral accuracy and availability. This suggests that place-level semantics and boundary-aware constraints are essential for preventing geometrically plausible but semantically invalid trajectories.

Removing the Landmark Observation Consistency and Visibility factors moderately degrades integrity and availability, demonstrating that even weak or relational semantic constraints contribute meaningfully to robustness under ambiguous or degraded sensing conditions.

In contrast, disabling the Speed-Limit Prior mainly affects longitudinal accuracy, while its impact on integrity remains limited. This indicates that motion-related semantic priors primarily stabilize local estimation rather than driving overall robustness gains.

## 6. Conclusions

This paper proposed a semantic-driven localization framework for robust vehicle localization in Level 4 autonomous driving environments. Unlike conventional approaches that rely primarily on geometric consistency, the proposed method incorporates semantic reasoning to preemptively eliminate logically and contextually invalid pose hypotheses and to selectively apply context-consistent constraints, reconstructing and localizing as a context-aware state inference problem.

Extensive experiments across diverse environmental conditions and sensor-failure scenarios demonstrate that the proposed framework maintains stable, reliable localization performance even in GNSS-degraded environments. In particular, semantic reasoning enables selective use of context-relevant sensor observations, suppressing unreliable measurements and facilitating structured, effective multi-sensor fusion. This selective sensor-fusion strategy prevents inconsistent constraints from affecting the optimization backend and enhances robustness against sensor degradation.

Overall, the results confirm that integrating semantic reasoning with selective sensor observation provides a practical and scalable solution for reliable localization in complex real-world environments. The proposed framework offers a strong foundation for future extensions toward cooperative perception and large-scale autonomous driving systems.

### Future Work

The proposed framework currently assumes the availability of HD maps and is evaluated under scenarios where such maps are accessible and periodically maintained, which inherently limits its applicability to environments that satisfy these conditions. Localization performance is therefore influenced by map quality, including geometric inaccuracies, semantic labeling errors, and inconsistencies arising from environmental changes. In the experimental scenarios considered in this work, global relocalization is not explicitly addressed; instead, the framework focuses on maintaining localization robustness and minimizing error accumulation under degraded sensing conditions within a fixed map reference. To relax these assumptions and improve general applicability, future work will investigate integrating lightweight map representations from open-source platforms such as OpenStreetMap [73] with dynamic, perception-driven map update mechanisms. In addition, extending the framework to support semantic-aware relocalization in partially mapped or map-inconsistent environments will be explored as an important direction for future research. Furthermore, cooperative perception and localization enabled by V2V communication will be explored to collaboratively refine semantic map information and compensate for outdated or incomplete map priors. These directions are expected to improve robustness to map imperfections and facilitate generalization of the proposed semantic reasoning framework beyond highly structured autonomous driving environments.

## Figures and Tables

**Table 1:** Map Node and Link Information.

Name	Info	
Node	ID	
	Point	
Link	ID	
	R_LinkID	
	L_LinkID	
	FromNodeID	
	ToNodeID	
	Points	
	RoadType	1: General road
		2: Tunnel
		3: Bridge
		4: Underground road
		5: Elevated highway

**Table 2:** Perceptible Object Attributes.

Object	Attribute	
Safety Sign	ID	
	Point	
Traffic Light	ID	
	Point	
Kilopost	ID	
	Point	
Speed Bump	ID	
	Point	
Surface Mark	ID	
	Points	
	MarkType	1: Arrow
		2: Crosswalk
		3: Stop Line

**Table 3:** Class of environmental elements based on TOSM.

Class			
Environmental Element	Object	StaticObject	Obstacle
		DynamicObject	Person
			Vehicle
			SignObject
		LandmarkObject	SurfaceMarkObject
			TrafficLightObject
			FacilityObject
	Place	HDPlace	RoadSegment
			Intersection
			BridgeSection
			TunnelSection
			EmergencyLane
		SemanticPlace	UnitPlace
		NonDrivablePlace	Sidewalk
			Crosswalk
			Restricted
	Robot	EgoVehicle	Car
		Sensor	Camera
			LiDAR
			Radar
			IMU
			GNSS

**Table 4:** Object properties for semantic HD map and reasoning.

Property	Domain	Range	Characteristics
***Spatial Relations***			
isNextTo	Place/Object	Place/Object	
isNearBy	Object	Object	symmetric
isInsideOf	Object/Place	Place	transitive
isConnectedTo	Place	Place	transitive
***Robot Relations***			
isLocatedAt	Robot	Place	
isMoveTo	Robot	Place	
isLookingAt	Robot	Object/Place	
isAlignedHeading	Robot	Place	
isOccupiedBy	Robot/Object	Place	
***Sensor Relations***			
isDetects	Sensor	Object	
isMeasureTo	Sensor	Object	
isReliable	Sensor	Place	
isEquipped	Sensor	Robot	
***Semantic Relations***			
isSameObject	Object	Object	symmetric
hasSemanticConsistency	Object	Place	
isContextualTo	Object	Place	
canMoveTo	Robot	Place	
isReachableTo	Robot/Place	Place	

**Table 5:** Datatype properties for object.

Property	Domain	Range
***Symbolic Model***		
Name	Object	string
ID	Object	int
***Explicit Model***		
pose	Object	(x, y, z, quaternion)
size	Object	vector
color	Object	(r, g, b)
velocity	DynamicObject	(μ, v, w, p, q, r)
descriptor	Object	vector
boundingBox	Object	(l, w, h)
semanticLabel	Object	string
measurementSource	Object	list
***Implicit Model***		
isKeyObject	Object	boolean
isMovable	DynamicObject	boolean
isStatic	StaticObject	boolean
isLandmark	LandmarkObject	boolean
isCandidate	Object	boolean
confidence	Object	float
stability	Object	float
lifetime	Object	float
trafficState	TrafficLightObject	int
laneState	SurfaceMarkObject	int

**Table 6:** Datatype properties for place.

Property	Domain	Range
***Symbolic Model***		
Name	Place	string
ID	Place	int
***Explicit Model***		
boundary	Place	polygon
coordinateFrame	Place	string
***Implicit Model***		
hasObject	Place	boolean
complexity	Place	float
sensorReliability	Place	list
candidate	Place	boolean
isLeafPlace	Place	boolean
isRootPlace	Place	boolean
speedLimit	RoadSegment	float
laneType	RoadSegment	int
direction	RoadSegment	int
priority	Intersection	string
isNotRestricted	Intersection	boolean
isOccupyed	UintPlace	boolean

**Table 7:** Datatype Properties for Robot.

Property	Domain	Range
***Symbolic Model***		
Name	Robot	string
ID	Robot	int
***Explicit Model***		
pose	Robot	(x, y, z, quaternion)
velocity	Robot	(μ, v, w, p, q, r)
limit	Robot	(μ, v, w, p, q, r)
size	Robot	(l, w, h)
***Implicit Model***		
types	Robot	string
hasGoal	Robot	(x, y, z, quaternion)
hasPlan	Robot	vector
capability	Robot	string
purpose	Robot	string
coverage	Sensor	(x, y, z, r, θ)
frequency	Sensor	float
isReliable	Sensor	boolean
isWorking	Sensor/Robot	boolean
state	Sensor/Robot	int

**Table 8:** Sensor configurations of the experimental vehicle platforms.

Category	Specification	Platform A	Platform B
Vehicle	Model	KIA Sportage	Hyundai IONIQ 6
Camera	Total number	4	6
	Coverage	360° field of view	360° field of view
	Frequency	10 Hz	10 Hz
	Model	Stereolabs ZED X × 2 Luxonis OAK-D-Pro × 2	e-con Systems STURDeCAM31 × 6
LiDAR	Total number	3	4
	Coverage	180° field of view	360° field of view
	Frequency	10 Hz	10 Hz
	Model	Hesai AT128 × 1 Scala Gen2 × 2	RS-LiDAR M1P × 3 RS128 × 1
Radar	Total number	4	–
	Coverage	360° field of view	–
	Frequency	10 Hz	–
	Model	Scala MCR1 × 4	–
RTK-GNSS	Model	SYNEREX MGI-2000	NovAtel PwrPak7D-E1
	Frequency	10 Hz	50 Hz
IMU	Model	HBK 3DM-GV7	HBK 3DM-GV7
	Frequency	100 Hz	100 Hz
Computing Unit	Total number	3	3
	Model	ADLINK DLAP-800X-DC/M16G × 1 NVIDIA Jetson AGX Orin × 2	ADLINK ASDSM8SHI-2TBT0 × 1 NVIDIA Jetson AGX Orin × 2
Summary	Total sensor	13	12

**Table 9:** Scenario-wise localization performance under nominal and degraded conditions.

Scenario	Accuracy			Reliability		Comments
	Lon (m)	Lat (m)	Heading (rad)	Integrity (PHMI/h)	Availability (%)	
**S1**	0.612	0.402	0.128	0.0502	99.12	Normal
**S2**	0.750	0.460	0.157	0.0720	98.21	GPS Interference
**S3**	1.043	0.545	0.197	0.0945	95.32	GPS Disruption
**S4**	0.920	0.500	0.180	0.0861	96.42	GPS Shadow
**S5**	0.980	0.570	0.190	0.0893	94.25	S4 + Lidar Fail
**S6**	1.048	0.517	0.175	0.0871	94.78	S4 + Camera Fail

**Table 10:** Overall localization results across defined experimental scenarios.

Attempt	Position Error (m)				Integrity (PHMI/h)			Availability (%)			Time (s)
	With GPS		Without GPS		Lon	Lat	Avg.	Lon	Lat	Avg.	
	Lon	Lat	Lon	Lat							
**Trial 1**	0.652	0.379	0.849	0.484	0.0197	0.143	0.0816	99.1	94.9	97.0	2500
**Trial 2**	0.667	0.392	0.807	0.434	0.0377	0.286	0.162	98.6	90.0	94.3	1244
**Trial 3**	0.637	0.366	0.891	0.535	0.00167	0.000456	0.00106	99.7	99.8	99.8	1257
**Trial 4**	0.450	0.549	0.902	0.690	0.143	0.177	0.160	95.8	94.8	95.3	1799
**Trial 5**	0.340	0.340	0.491	0.472	0.000296	0.000370	0.000333	99.9	99.9	99.9	1062
**Average**	0.549	0.405	0.788	0.523	0.0405	0.121	0.0810	98.6	95.9	97.3	–

**Table 11:** Comparison of localization performance under GPS shadowing conditions.

Method	Lon (m)	Lat (m)	Notes
EKF [30]	2.852	1.942	Sensor fusion
UKF [70]	2.431	1.753	Sensor fusion
LeGO-LOAM [9]	1.627	0.982	Lidar SLAM
HDL Graph SLAM [64]	**1.482**	**0.912**	Lidar SLAM
Generalized ICP (Genz-ICP) [16]	1.954	1.123	Lidar SLAM
OpenVINS [71]	2.103	1.300	Visual SLAM
Stella VSLAM [72]	1.881	1.222	Visual SLAM
**Proposed Method**	**1.143**	**0.745**	

**Table 12:** Comparison of localization performance with and without cooperative perception.

Sequence	Method	Scenario	Lon (m)	Lat (m)	Notes
A	Independ	1	0.971	0.562	Baseline
B	Independ	1	1.749	1.353	Baseline
A	Cooperative	4	1.020	0.632	Object sharing
B	Cooperative	4	1.836	1.403	Object sharing
A	Cooperative	6	0.948	0.551	Only uses B’s objects
B	Cooperative	6	1.081	1.132	Only uses A’s objects

**Table 13:** Busan semantic object statistics.

Object Layers	Count
Traffic lights	388
Kiloposts	34
Safe signs	958
Semantics	4545
Objects	5925
Nodes	1116
Lanes	1969
Height barriers	19
Vehicle protections	277
Sections	42
Speedbumps	3
Surface marks	668

**Table 14:** Pangyo Semantic Object Statistics.

Object Layers	Count
Traffic lights	320
Kiloposts	0
Safe signs	445
Semantics	411
Objects	1176
Nodes	1433
Lanes	2189
Height barriers	12
Vehicle protections	483
Sections	11
Speedbumps	0
Surface marks	597

**Table 15:** Comparison of localization performance with and without semantic rules.

Method	Longitudinal Error (m)				Lateral Error (m)			
	Mean	Max	Min	Var	Mean	Max	Min	Var
Without Rules	1.842	3.912	0.954	0.812	1.102	2.531	0.582	0.497
With Rules	1.128	2.104	0.611	0.421	0.744	1.320	0.311	0.238

**Table 16:** Computational performance comparison with and without semantic rules.

Nodes	Total Factors	Relinearization		Avg Time (ms)		Max Time (ms)	
		Without Rules	With Rules	Without Rules	With Rules	Without Rules	With Rules
9142	100,552	1760	16	13.331	1.126	271.155	10.111
13,875	152,615	2560	17	21.653	2.456	426.304	15.806
18,634	204,964	3571	19	31.208	3.903	608.295	21.928
24,118	265,288	4497	21	42.437	5.669	791.743	29.847

**Table 17:** Ablation study on semantic constraints in global optimization.

Configuration	Accuracy		Reliability	
	Lon. Err. (m)	Lat. Err. (m)	Integrity (Avg.)	Availability (%)
Proposed Semantic-Aware Optimization (Full)	0.562	0.415	0.079	94.98
No Outlier Rejection	2.000	0.670	0.414	81.97
No Place-Consistency and SDF Constraint	0.686	1.395	0.109	82.38
No Speed-Limit Prior	1.102	0.506	0.092	84.02
No Landmark Observation Consistency	0.864	0.812	0.172	88.44
No Landmark Visibility Constraint	0.653	0.540	0.083	84.62
No Landmark Factor	3.833	3.228	2.505	57.49
Raw Sensor Fusion Baseline (No Semantic Constraints)	5.755	5.017	3.716	45.98

## Data Availability

The data presented in this study are not publicly available due to project confidentiality agreements. Further inquiries can be directed to the corresponding author.
